# A neurally plausible schema-theoretic approach to modelling cognitive dysfunction and neurophysiological markers in Parkinson's disease

**DOI:** 10.1016/j.neuropsychologia.2020.107359

**Published:** 2020-03-16

**Authors:** Andrea Caso, Richard P. Cooper

**Affiliations:** Birkbeck, University of London, UK

**Keywords:** Parkinson's disease, Neurocomputational model, ERP, Basal ganglia, Cognitive control, Wisconsin card sorting test

## Abstract

The cognitive mechanisms underlying sequential action selection in routine or everyday activities may be understood in terms of competition within a hierarchically organised network of action schemas. We present a neurobiologically plausible elaboration of an existing schema-based cognitive model of action selection in which the basal ganglia implements an activation-based selection process that mediates between assumed cortical representations of rule-based schemas. More specifically, the model employs a network of basal ganglia units with computations performed by individual BG nuclei, embedded in a corticothalamic loop that disinhibits schemas according to the received feedback. We provide bridging assumptions for linking the operation of the model with ERP components that describe the error-related negativity (ERN) and the parietal switch positivity (PSP), and evaluate the model against behavioural and neural markers of performance of the Wisconsin Card Sorting Test by healthy control participants and Parkinson's Disease patients.

## Highlights

• A neuro-computational model of schema-based action selection is presented.
• The model includes cortical and striatal learning mechanisms.
• Parkinson’s Disease is simulated via a decreased learning rate in the striatum.
• The model accounts for effects of Parkinson’s Disease on WCST performance.
• The model also reproduces key effects of Parkinson’s pathology on ERP signals.

## 1 Introduction

In an influential account of the control of thought and action, Norman and Shallice (1986) drew a distinction between routine or over-learned behaviours and non-routine behaviours. Routine behaviour, they argued, reflects the enactment of learned schemas, via a system they called *contention scheduling*, while non-routine behaviour was held to reflect the operation of a deliberative system, the *supervisory system*, that operates on behaviour indirectly by selectively biasing the representations of schemas within contention scheduling. The account was motivated by both the slips and lapses in action of neurologically healthy participants (e.g., Norman, 1981; Reason, 1979, 1984) and the action errors of neurological patients (e.g., Duncan, 1986; Lhermitte, 1983; Luria, 1966).

A basic premise of the contention scheduling/supervisory system framework is that much of everyday action (and thought) is schema-based. At a relatively low level, consider the steps involved in changing down a gear when driving a manual car and slowing for traffic lights or for a sharp corner. One must first engage the clutch with the left foot, then use the gear stick to deselect the current gear and select the new (lower) gear while simultaneously touching the accelerator with the right foot to match the engine revs to the gear selection, and then slowly release the clutch. A critical part of learning to drive a manual car is automating these steps into a single routine — an action schema — that can be performed as a single unit, seemingly without conscious or deliberate control of each step.

Schemas are also held to organise routine behaviour at higher levels. Consider the morning routine, and specifically preparing breakfast. While performance of the activities involved is subject to the specifics of the environment (and so any two instances of breakfasting, are not identical), the subcomponents for any individual often remain relatively fixed (e.g., preparing coffee and cereal, with each having subcomponents, such as, in the case of preparing cereal: locating a bowl, the cereal box, milk and a spoon, and then pouring cereal and milk into the bowl). Similar arguments apply for other everyday behaviours, such as dressing and grooming, or commuting, or the evening routine and its elements.

The schemas referred to in the previous paragraphs have a number of critical properties. First, like schemas in other domains (e.g., memory, language) they are structured abstractions over instances of specific items. Thus, the schema for changing down gears in a manual car is an abstraction over many instances of the behaviour, and while each instance involves a specific sequence of actions performed in a specific vehicle at a specific moment in time, the abstract schema does not refer to such specifics.

A second key property of action schemas is that they are hierarchically structured. That is, higher-level schemas (like preparing cereal) consist of partially ordered sets of lower-level schemas (the components listed above), and those lower-level schemas may themselves consist of partially ordered sets of even lower-level schemas (such as visually searching for an object, grasping an object, etc.). Higher-level schemas are more temporally extended in nature than their component schemas, and schemas at each level may occur in any number of super-ordinate schemas (so locating a bowl might be involved in several higher-level schemas such as preparing breakfast cereal or preparing soup).

This view of action, as being constructed from instances of schemas, raises the question of how schemas might be selected from the pool of those known to an agent and then instantiated in order to control action. This is the function of contention scheduling. The contention scheduling theory proposes that representations of schemas, such as those described above, compete for the control of action on a moment-by-moment basis. Representations may be triggered or partially activated by learned contingencies in the environment (e.g., the presence of a red-light or a sharp bend ahead when driving) or by excitation from higher-level schemas.^1^ Contention scheduling selects between schemas by combining these sources of excitation (environmental or bottom-up triggering and top-down excitation), with the most active schema or schemas (above a threshold) being selected and thence controlling behaviour. Critically, the supervisory system operates not by directly selecting actions but by constructing temporary control structures (i.e., temporary schemas) which bias schema representations within contention scheduling by exerting top-down excitation. Pathologies of action are held to arise when this biasing fails or is inappropriately applied, or as a result of dysfunction in the flow of activation within contention scheduling.

Cooper and Shallice (2000) provided an interactive activation model of the contention scheduling system and demonstrated that, when lesioned in theoretically motivated ways, the model was able to produce disturbances of action selection that were qualitatively analogous to those of patients showing various pathologies of sequential action (specifically, those of Schwartz et al., 1998, 1995; Schwartz et al., 1991). Cooper and Shallice (2000) also argued that the model could account for utilisation behaviour — a tendency to use objects in the immediate environment in object-appropriate ways, despite instruction and apparent intention to the contrary (e.g., Boccardi et al., 2002), through either increased bottom-up or decreased top-down excitation, and the bradykinesia of Parkinson's Disease, through either increased lateral inhibition or decreased self activation within the schema network. Moreover, in subsequent work the model was shown to be capable of producing the quantitative error profiles of Action Disorganisation Syndrome (Cooper et al., 2005) (resulting from frontal injury in patients and following from addition of noise to activations in the schema network in the model) and Ideational Apraxia (Cooper, 2007) (resulting from damage to left temporo-parietal cortex in patients and following from disconnection between the representations of schemas and objects in the model).

Despite these neuropsychological links, the model was agnostic with regard to the neural bases of the subprocesses of schema selection. For example, it accounted for bradykinesia (slowed action initiation) in Parkinson's Disease (PD) patients purely in terms of an imbalance between self activation and lateral inhibition within an interactive activation network in which nodes corresponded to schemas. No lower-level mechanistic account of these processes was provided, beyond pointing to the possible involvement of dopamine in regulating competition, and no attempt was made to relate the model to executive function deficits known to be associated with Parkinson's Disease, such as deficits relating to set shifting, inhibition, and selective attention (see Kudlicka et al., 2011, for a metareview), or to increased impulsivity in medicated Parkinson's patients (Martini et al., 2018).

Moreover, the original model did not learn, either at the cortical or the subcortical level, yet there is brain-based evidence for learning within the action selection system at both levels. For example, several imaging studies have shown that, when learning a sequential task, prefrontal cortical activity declines as the task becomes more well-practiced (e.g., Jenkins et al., 1994). Raichle et al. (1994) found similar effects of learning on prefrontal activation in a non-motor (verbal learning) task. These studies suggest that prefrontal cortex is involved in the generation and active maintenance of temporary schemas. Other studies have shown involvement of the basal ganglia and more specifically the dopamine system in the learning of action-related tasks and cognitive skills (e.g., Poldrack et al., 1999; Schultz et al., 1993). Montague, Dayan, and Sejnowski (1996) interpret such findings as evidence for the dopaminergic encoding of a reward-prediction error (i.e., the difference between anticipated and received reward) within the basal ganglia. Consistent with this, subsequent imaging work within a reinforcement learning theoretical framework (Sutton and Barto, 1998) has found high reward prediction error to be associated with activity in the striatum while high state prediction error (i.e., large differences between anticipated and observed states of the environment) has been associated with prefrontal activation (e.g., Gläscher, Daw, Dayan, & O'Doherty, 2010). From the perspective of the contention scheduling/supervisory system theory, this evidence suggests that the creation and active maintenance of temporary schemas is probably performed within frontal neocortical tissue, while subcortical learning would involve tuning the selection of appropriate schemas according to their prior reinforcement history. This cortical/subcortical distinction is quite coarse, and it may only apply to motor programmes that are ontogenetically and phylogenetically more recent, as there is also evidence for action selection mechanisms in the brainstem (Humphries et al., 2007).

In the remainder of this paper we present an elaboration of the Cooper and Shallice model, in which activation-based selection processes are implemented by a neurobiologically plausible model of the basal ganglia that mediates between assumed frontal representations of schemas. Critically, the model includes learning mechanisms both within the cortical and basal ganglia components. We illustrate the model within the context of the Wisconsin Card Sorting Test (WCST) as used by Lange, Seer, et al. (2016), who tested healthy participants and PD patients in the so-called “Madrid” version of the task (Barcelo, 2003). It is data from this work that we use to evaluate the extended model. Our model demonstrates how the WCST can be performed by a hierarchically organised set of schemas. Higher-level schemas in the model correspond to the three sorting rules required for successful completion of the task, while lower-level schemas correspond to sensorimotor procedures for placing cards at each of the various target locations (see Cooper, 2009). In constrast to earlier work, instead of using mutual lateral inhibition among the schemas, the model employs a network of basal ganglia (BG) units with computations performed by individual BG nuclei, as described by Gurney et al. (2001), embedded within corticothalamic loops that disinhibit schemas according to the received feedback. Performance within the model is controlled by parameters that alter the relationship between schemas without altering their content. We analyse how the main parameters affect errors in performance and how variation of their values simulates the type of performance seen in patients with Parkinson's Disease. Then, we relate the internal variables of the model to two ERP components (ERN and PSP) that have been observed in the WCST task by Lange, Seer, et al. (2016), and which are considered indices of conflict detection and set-shifting, respectively. This allows the identification of the internal computational processes that give rise to these ERP components and allows the teasing out of the contribution of the basal ganglia to these frontostriatal processes. The model thus constitutes a bridge between neural and behavioural data.

## 2 An illustrative task: the Wisconsin Card Sorting Test

While the model is intended to illustrate the general mechanisms of schema competition and selection as mediated by the basal ganglia, we ground it here in a specific task, namely the Wisconsin Card Sorting Test (WCST), and specifically the version described by Barceló (2003) and subsequently used in several studies, including those of Lange, Seer, et al. (2016), with PD patients and age-matched controls. This task was chosen for several reasons. Firstly, as with the standard WCST, it exercises hierarchical schema-based control, in the sense that successful performance of the task requires simultaneous selection of a higher-level schema (e.g., sort the cards according to the colour of the images on them) and lower-level schemas (e.g., pick up or select the card to be sorted and place or drag it to beneath the matching target card). Secondly, Lange, Seer, et al. (2016) provide detailed behavioural and ERP data for PD patients and age-matched controls. Thirdly, that data shows between-group differences (in both behavioural and ERP measures) that have previously been attributed at the neural level to differences in sub-cortical dopamine concentration between the two groups and at the cognitive level to differences in conflict-detection and set-shifting behaviour. Finally, with the addition of bridging assumptions linking model variables to ERPs, it is possible to simulate these ERP components within the model.

### 2.1 The WCST of Barceló (2003)

**Fig. 1:**
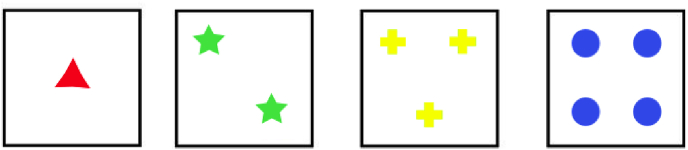
The four target cards used in the Wisconsin Card Sorting Test.

Within the standard Wisconsin Card Sorting Test, participants are required to sort a series of cards into four categories based on binary (i.e., correct/incorrect) feedback. Each card shows one, two, three, or four identical shapes (triangle, star, cross, or circle), printed in one of four colours (red, green, yellow, or blue), as shown in Fig. 1. It is therefore possible to sort cards according to the colour, number or shape of images on the cards. To succeed at the task, participants must match each successive card with one of four target cards, and use the subsequent binary feedback to discover the appropriate sorting rule (i.e., sort by colour, number or shape). Once they have discovered the rule they should continue applying it for as long as they continue receiving positive feedback, but the experimenter periodically changes the rule without notice — in most versions after 10 successive correct responses. The participant then has to discover and adapt to the new rule. The task thus assesses multiple abilities, including hypothesis generation, task set maintenance, and cognitive flexibility.

There are several differences between the standard WCST as described in the manual of, for example, Heaton (1981) and the version employed by Barceló (2003) and Lange, Seer, et al. (2016) and modelled here. Firstly, in the standard version participants must infer the potential sorting rules, while in the latter version participants are explicitly told that they should choose between the three sorting rules. This removes a degree of complexity from the task. Secondly, in the standard version of the task the deck of stimulus cards includes all possible combinations of features (colour, shape, number — 64 combinations in total). This means that stimulus cards can match target cards on more than one feature. For example, a stimulus card might show two blue circles, which would match the second target card in Fig. 1 on one feature but the fourth target card on two other features. In the Barceló (2003) variant of the task this stimulus ambiguity is removed. Only the 24 cards that match a single target card feature are included in the deck. This removes ambiguity both from the feedback to participants and in scoring participant responses.^2^

### 2.2 Dependent measures and key relevant findings

The WCST is a complex task that generates multiple dependent measures. Perhaps the simplest of these is response time (RT) — the time taken to sort each stimulus card. While the WCST is not traditionally a timed test, Lange, Seer, et al. (2016) report response times for both healthy control and PD participants, and show systematic differences with respect both to type of trial and to group. Thus both healthy controls and PD participants were slower on trials requiring a rule change than on trials not requiring a rule change, and PD participants were slower on all types of trial than healthy age-matched controls, but the two factors (trial type and group) did not interact.

More generally, the variables of interest in the WCST are the frequencies with which various types of errors (i.e., responses leading to negative feedback) are made by participants. Following Lange, Kröger, et al. (2016), we consider three types of errors that might be made on each card sorting trial: perseverative errors (PE), set loss errors (SL), and integration errors (IE). A perseverative error (PE) is scored when a participant's response is incorrect but is consistent with the previously successful rule, despite negative feedback on the previous trial. A set loss (SL) error is scored when the participant's response indicates a change of sorting rule despite having received positive feedback on the previous trial. An integration error (IE) is scored when a participant's response indicates a change of sorting rule following negative feedback, but when the new sorting rule adopted by the participant could have been ruled out by feedback on the trial before.

Thus, perseverative errors occur when the participant fails to switch rules despite negative feedback, perhaps because either the feedback is ignored or inhibition of the currently selected rule is insufficient. In contrast, set loss errors may be due to loss of the mental representation of the current rule, to a diminished reward sensitivity (reflecting, for example, a form of habituation) or, as we show below, to the lack of stability of sensorimotor schemas, which in turn allows a stimulus to drive the response. Finally, integration errors, at least as argued by Lange, Kröger, et al. (2016), reflect a failure in remembering either recently tried rules or previous negative feedback.

Consistent with a previous meta-analysis (Kudlicka et al., 2011), Lange, Seer, et al. (2016) found that their PD patients made more perseverative errors than their healthy age-matched controls. PD patients also made more set loss errors than the controls, but not more integration errors (see Lange et al., 2017).

A key advance in the work of Lange, Seer, et al. (2016) was the reporting of ERP components related to WCST performance. They focused in particular on two components, the P3a — a positive fronto-central potential occurring approximately 250–280 msec after presentation of a stimulus, and whose amplitude is thought to reflect attentional orienting — and the SPP (Sustained Parietal Positivity, also known as the PSP: Posterior Switch Positivity) — a more posterior positive component occurring late in processing and held to reflect set-shifting. Lange, Seer, et al. (2016) argued that the amplitudes of these ERP components should correlate with corresponding behavioural measures. In particular, they found (across their entire sample, including PD participants and controls) that the amplitude of the P3a correlated (negatively) with the proportion of perseverative errors, perhaps indicating that stronger attentional orienting results in more successful set switching, and that the amplitude of the SPP correlated (negatively) with the proportion of set loss errors, suggesting that stronger SPP reflects better set maintenance.

Given these findings, and the well-known basal ganglia pathology arising from PD (see Gale et al., 2008, for a review), the WCST is therefore a highly appropriate task for evaluating a model of schema selection that incorporates basal ganglia function.

## 3 A neurobiologically-plausible model of schema selection

### 3.1 Overall architecture

**Fig. 2:**
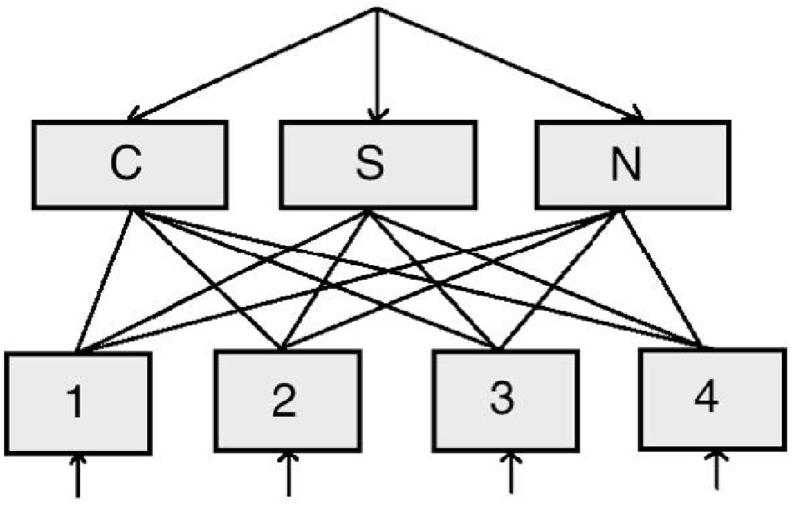
Schema nodes within the model. The top three (cognitive) schemas represent the sorting rules (C: colour, S: shape, N: number) while the bottom four (sensorimotor) schemas represent the acts of placing a stimulus card below one of the four target cards.

The model consists of two sets of schema nodes (see Fig. 2) and, for each schema node, a set of basal ganglia units as described below. Each node/unit has an associated activation value which varies over time as a function of excitation and inhibition received from other nodes/units in the model. One set of schema nodes corresponds to the sorting rules — one node each for *sort by colour*, *sort by shape* and *sort by number*. The second set of schema nodes corresponds to sensorimotor schemas for placing stimulus cards below target cards. As there are four target cards (Fig. 1), there are four sensorimotor schema nodes. We refer to the sorting schemas as cognitive because it is assumed that they receive excitation from the supervisory system. In contrast, the sensorimotor schemas receive a signal when a stimulus is presented, while their activation entails selection of a motor response.

**Fig. 3:**
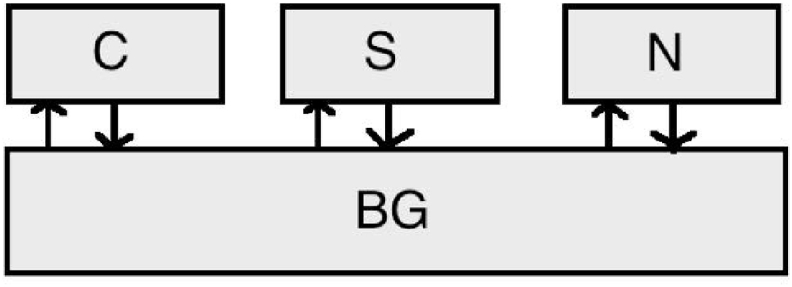
Schematic of the connections between cortical schema units and the basal ganglia (C: colour, S: shape, N: number). The sensorimotor units have analogous connections with the basal ganglia.

**Fig. 4:**
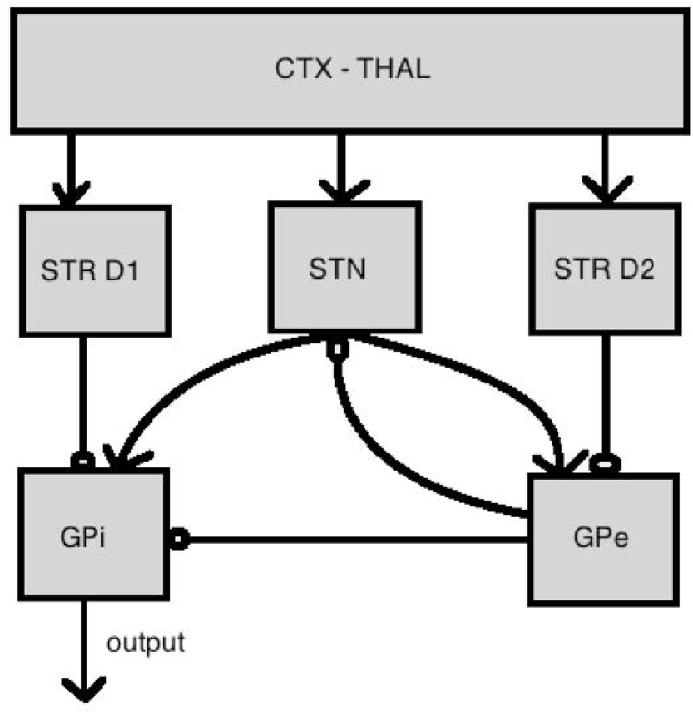
Basal ganglia units associated with each schema node (for both cortical and sensorimotor schemas). (CTX-THAL: corticothalamic complex/input of the model; STR D1: Striatum with D1 modulation; STN: Subthalamic Nucleus; STR D2: Striatum with D2 modulation; GPe: Globus Pallidus external segment; GPi: Globus Pallidus internal segment.)

Each set of schema nodes feeds into and receives output from a basal ganglia “layer”, as shown in Fig. 3 (for cognitive schemas), where the basal ganglia layer consists of five units for each schema node, as shown in Fig. 4. Thus, each schema node participates in a simulated cortico-subcortical loop. The basal ganglia units and their interconnectivity are based on our understanding of the functional biology of the basal ganglia (Gurney et al., 2001). The baseline activity of the basal ganglia and thalamic complex acts to suppress cortical activity (Wichmann and DeLong, 1996), which is represented by cognitive and sensorimotor schemas in our model. The joint action of the basal ganglia units registers the activity in all schema nodes and, while suppressing the activation of most of them, it partially or totally disinhibits one or a few of them, thereby increasing their chances of selection. The computation occurs in units corresponding to the caudate and putamen (*str* subscript), the subthalamic nucleus (*stn* subscript), the globus pallidus external segment (*gpe* subscript) and the globus pallidus internal segment (*gpi* subscript).

### 3.2 The cortical component

#### 3.2.1 Activation calculation

For both cognitive and sensorimotor schemas, activation is calculated at successive time-steps according to three equations (see Equation (1) for cognitive schemas and Equation (2) for sensorimotor schema nodes). In each case, the first equation determines the strength of the input ($u_{i}^{t}$) to node *i* at time *t*, the second implements a simple low-pass filter that evens out the input signal over time, and the third applies the logistic function σ, with parameters that determine its gain or slope α and its threshold β, to constrain the node's activation to between zero and one.

*Cortex (Cognitive Schemas):* The three cognitive schema nodes are assumed to receive a constant excitatory signal, $o_{ext}$, plus input from their corresponding thalamus unit in the basal ganglia (see below):

(1) $$\begin{array}{l} u_{i}^{t}\leftarrow o_{ext}+o_{thal,i}^{t-1}\\ a_{i}^{t}\leftarrow \delta \cdot a_{i}^{t-1}+\left(1-\delta \right)\cdot u_{i}^{t}\\ o_{i}^{t}\leftarrow \sigma_{\beta_{pfc},\alpha_{pfc}}\left(a_{i}^{t}\right) \end{array}$$

*Cortex (Sensorimotor Schemas):* The four sensorimotor schema nodes are assumed to receive input from cognitive schema nodes (scaled by $w_{i,j}^{rule}$, the weight of connection from each cognitive schema node *j* to each sensorimotor schema node *i*), plus $o_{stim}$ if a stimulus card is present and matches the corresponding target card on any feature (so a card showing two red crosses will activate the first, second and third sensorimotor schema units; see Fig. 1), plus input from the corresponding thalamus unit in the basal ganglia:

(2) $$\begin{array}{l} u_{i}^{t}\leftarrow \underset{j}{\sum }w_{i,j}^{rule}o_{ctx,j}^{t}+o_{stim}+o_{thal,i}^{t-1}\\ a_{i}^{t}\leftarrow \delta \cdot a_{i}^{t-1}+\left(1-\delta \right)\cdot u_{i}^{t}\\ o_{i}^{t}\leftarrow \sigma_{\beta_{sma},\alpha_{sma}}\left(a_{i}^{t}\right) \end{array}$$

For the third clause in Equation (1) and Equation (2) above (and all other equation sets below), $\sigma_{\beta ,\alpha }$, often referred to as the logistic function, is given by Equation (3):

(3) $$\sigma_{\beta ,\alpha }\left(x\right)=\frac{1}{1+e^{-\alpha \cdot \left(x-\beta \right)}}$$

#### 3.2.2 Schema selection

A schema is selected when its activation exceeds a static threshold $\theta_{s}$ and the area below the activation value from the last trial reaches a threshold $\theta_{A}$, where both $\theta_{s}$ and $\theta_{A}$ are parameters of the model. The former reflects the possibility that no schema may be selected, resulting in no action. The latter reflects the assumption of integrators that accumulate data until a decision can be made (Forstmann et al., 2016). Only selected cognitive schemas pass excitation to sensorimotor schemas. Thus $w_{i,j}^{rule}$ in Equation (2) is zero for non-selected cognitive schemas. Selected sensorimotor schemas trigger corresponding motor acts (i.e., placement of the stimulus card under the target card corresponding to the selected sensorimotor schema).

#### 3.2.3 Cortical learning

We assume that the slope or gain of the saturation functions of cortical units dynamically adapts to the level of conflict between those units. In particular, when the activation of several cortical representations is very similar, and the basal ganglia alone cannot arbitrate between different representations because feedback/reward has not yet been received and computed, a mechanism is required to resolve this conflict and make a decision. Moreover, the stability of cognitive representations needs to be sensitive to the need to trade off exploration and exploitation at different levels of the schema hierarchy (Goschke and Bolte, 2014). Allowing the gain of the activation function at each level of the hierarchy to vary in response to conflict provides a mechanism for this.

Here, we implement a mechanism that allows the cortical sensorimotor nodes to change the gain of their saturation function $\alpha_{sma}$ via the free parameter $\varepsilon_{sma}$ according to Equation (4):

(4) $$\alpha_{sma}\leftarrow \left(1+\zeta_{sma}\right)\overset{N}{\underset{i}{\prod }}\left(1+\varepsilon_{sma}+o_{sma,i}\right)$$

where $\zeta_{sma}$ is the sensorimotor unit noise and the product is over all sensorimotor units (so *N* is 4 in the model of WCST). For simplicity we do not include the analogous dynamic slope adjustment for cognitive schema nodes.

Psychologically, increasing the gain is akin to reducing hesitancy in responding, given the same level of evidence. Equation (4) has the desirable property of a conflict construct, as illustrated by Berlyne (1957) (see also Botvinick et al., 2001). Thus, conflict should increase with the number and the activation value of competing representations, and it should peak when all activations peak. These criteria can be met by an infinite number functions, but a simple solution is provided by the product of activation values. Conflict should drive change to the schema activation values so as to stabilise or destabilise them as a function of their input, and this is therefore implemented through the change of slope of the saturation function in the sensorimotor units. Computationally, cognitive control acts by first detecting processes that make performance suboptimal and then adjusting control by changing attentional focus. This mechanism can carry out the stabilisation of the activation of any of the four sensorimotor schemas, pulling their activation to either side of the threshold value more quickly. The value of $\alpha_{sma}$ is updated each time feedback is given.

### 3.3 The basal ganglia component

#### 3.3.1 Activation calculation of basal ganglia units

Activation of basal ganglia nodes is calculated in a way analogous to activation of cortical units. Thus, in each case input, $u^{t}$, at time *t* is calculated. This is then smoothed using a weighted average and fed to a saturation function, which in all cases is the standard sigmoid function (Equation (3)) with parameters defining threshold and slope. Connectivity of basal ganglia units (and hence input to each unit) is as shown in Fig. 4.

All striatal channels receive a copy of the signal from the cortex, both in the cognitive (higher order) and the sensorimotor (lower order) loops, therefore the subscript ctx in the equations below indicates either cognitive or sensorimotor schemas.

*Striatum (STR D1 and STR D2):* The input to each striatal unit is just the current activation of the corresponding cortical unit:

(5) $$\begin{array}{l} u_{i}^{t}\leftarrow o_{cxt,i}^{t}\\ a_{i}^{t}\leftarrow \delta \cdot a_{i}^{t-1}+\left(1-\delta \right)u_{i}^{t}\\ o_{i}^{t}\leftarrow \sigma_{\beta_{str},\alpha_{str}}\left(a_{i}\right) \end{array}$$

*Subthalamic Nucleus (STN):* Subthalamic Nucleus units receive excitatory input from the cortex (weighted by $w_{stn}$) and inhibitory input from the External Segment of the Globus Pallidus (weighted by $w_{gpe,stn}$):

(6) $$\begin{array}{l} u_{i}^{t}\leftarrow w_{stn}o_{ctx}^{t}+w_{gpe,stn}o_{gpe,i}^{t-1}\\ a_{i}^{t}\leftarrow \delta \cdot a_{i}^{t-1}+\left(1-\delta \right)u_{i}^{t}\\ o_{i}^{t}\leftarrow \sigma_{\beta_{stn},\alpha_{stn}}\left(a_{i}\right) \end{array}$$

*Globus Pallidus External Segment (GPe):* Units in the External Segment of the Globus Pallidus receive excitatory input from the Subthalamic Nucleus (weighted by $w_{stn,gpe}$) and inhibitory input from the D2 channel of the Striatum (weighted by $w_{strD2,gpe}$):

(7) $$\begin{array}{l} u_{i}^{t}\leftarrow w_{stn,gpe}\sum_{j}o_{stn,j}^{t}+w_{strD2,gpe}o_{strD2,i}^{t-1}\\ a_{i}^{t}\leftarrow \delta \cdot a_{i}^{t-1}+\left(1-\delta \right)u_{i}^{t}\\ o_{i}^{t}\leftarrow \sigma_{\beta_{gpe},\alpha_{gpe}}\left(a_{i}\right) \end{array}\;$$

*Globus Pallidus Internal Segment (GPi):* Units in the Internal Segment of the Globus Pallidus receive excitatory input from the Subthalamic Nucleus (weighted by $w_{stn,gpi}$) and inhibitory input from the Globus Pallidus External Segment (weighted by $w_{gpe,gpi}$) and the D1 channel of the Striatum (weighted by $w_{strD1,gpi}$):

(8) $$\begin{array}{l} u_{i}^{t}\leftarrow w_{stn,gpi}\underset{j}{\sum }o_{stn,j}^{t}+w_{gpe,gpi}o_{gpe,i}^{t}+w_{strD1,gpi}o_{strD1,i}^{t}\\ a_{i}^{t}\leftarrow \delta \cdot a_{i}^{t-1}+\left(1-\delta \right)u_{i}^{t}\\ o_{i}^{t}\leftarrow \sigma_{\beta_{gpi},\alpha_{gpi}}\left(a_{i}\right) \end{array}$$

In the summation here, the index *j* ranges over all competing inputs from the Subthalamic Nucleus. This ensures that the basal ganglia units scale their interactions with each other and their outputs according to the global input signal, ultimately in order to appropriately promote competition among the cortical units.

*Thalamus (THAL):* Finally, units in the Thalamus receive input from the Globus Pallidus Internal Segment:

(9) $$\begin{array}{l} u_{i}^{t}\leftarrow o_{gpi,i}^{t}\\ a_{i}^{t}\leftarrow \delta \cdot a_{i}^{t-1}+\left(1-\delta \right)u_{i}^{t}\\ o_{i}^{t}\leftarrow -\sigma_{\beta_{thal},\alpha_{thal}}\left(a_{i}\right) \end{array}$$

The negation in the last clause of Equation (9) reflects the fact that the thalamus is tonically active and disinhibited by the basal ganglia complex.

The model contains three corticobasal pathways (see Fig. 4). These are traditionally called the direct, indirect, and hyperdirect pathways (Alexander et al., 1986). The direct pathway projects from the striatum directly into the globus pallidus (internal segment), while the indirect one passes through the external segment of the globus pallidus and the subthalamic nucleus. The hyperdirect pathways does not pass through the striatum at all, and reaches the globus pallidus (internal segment) directly from the cortex. As in the model by Gurney et al. (2001), direct and indirect pathways can be renamed according to their functionality, to selection and control pathways, respectively, in that action selection is mainly executed by the selection pathway, whilst the control pathway scales the output and thus affects the overall selection threshold.

#### 3.3.2 Basal ganglia learning mechanism

The basal ganglia units are regulated in a different fashion from the cortical nodes. While cortical nodes are solely regulated by their online state, regardless of history of activation and external stimuli, basal ganglia units change their characteristics with a history-based and reward-driven time course. This is reflected by adjusting $\beta_{str}$, the threshold of the saturation function in striatal units, which is assumed to be related to the level of striatal dopamine. A mechanism that alters this threshold as a function of current feedback and past history of activation in the respective cortical units is shown in Equation (10):

(10) $$\beta_{str,i}\leftarrow \left(\beta_{str,i}-\varepsilon_{str}\delta_{i}\right)\cdot \left(1+\zeta_{str,i}\right)$$

where the calculated value of $\beta_{str}$ is clipped to within the range [0, 1] if it falls below 0 or above 1.

In this equation, $\delta_{i}$ is the reward prediction error (RPE), expressed as the difference between the current feature matching value $f_{i}$ and the median activation value in the last trial $a_{i}$ (Equation (11)):

(11) $$\delta_{i}\leftarrow r_{i}\cdot \left(f_{i}-a_{i}\right)$$

where $r_{i}$ is either +1 or −1, according to whether the feedback is positive (correct response) or negative (incorrect response). This allows the model to bias $\beta_{str}$ in the correct direction.

In order to calculate the striatal saturation threshold, the feature match, $f_{i}$, is assigned to each cognitive schema according to Equation (12):

(12) $$f_{i}\leftarrow \{\begin{array}{cc} \left(2w_{neg}-1\right)-\left(m_{r}\cdot f_{i}^{t-1}\cdot r_{i}^{t-1}\right) & \text{if no matching feature}\\ +1 & \text{if at least one matching feature} \end{array}$$

where $m_{r}$ is a parameter that determines the extent to which feedback from previous trials affects the calculation.

If the correct rule matches the given response, $f_{i}$ assumes a value of +1. Otherwise, if $w_{neg}$ is 0 and $m_{r}$ is 0, the resulting value is −1, but increasingly higher values of $w_{neg}$ correspond to decreased negative reward sensitivity, while increasingly higher values of $m_{r}$ result in persistence of feedback from the previous trial. The RPE can therefore assume positive or negative values; for instance, if the external feedback is incorrect (the model selected the wrong card), the target card matches two features, and the schema has a high median activation value during the last trial, then the RPE will be negative but of small absolute value.

The mechanism defined by equations (10), (11), (12) tends to bias the activation for one of the three cognitive schema nodes through the basal ganglia units, as a function of a) the reward/feedback received, b) the immediately past value of $\beta_{str}$ (which is updated each time feedback is given) and c) the learning parameter $\varepsilon_{str}$. This generally results in selecting the action that has received the most immediate positive feedback, as in reinforcement learning algorithms (Sutton and Barto, 1998). Note that the parameter $\beta_{str}$ varies for the cognitive cortical units only. It is assumed that lower level actions represented in the sensorimotor schemas are not reinforced as strongly as higher-order actions, as stimuli are distributed randomly and so no sequence is discernible.

### 3.4 Parameters of the model

The full set of model parameters and their default values are shown in Table 1. The parameters specify: the strength of input to schema nodes ($o_{ext}$ and $o_{stim}$); the strength of connections between units (*w*); the slope (α) and threshold (β) of saturation functions; the smoothing constant in saturation functions (δ); learning rates for the striatum ($\varepsilon_{str}$) and for sensorimotor schemas ($\varepsilon_{sma}$); thresholds for schema selection (θ); and noise (ζ).

**Table 1:** The complete list of model parameters and their default values.

Parameter	Default Value	Description
δ	0.60	Smoothing constant in all integrations
$o_{ext}$	0.75	External input to cognitive schema units
$o_{stim}$	0.50	Input to sensorimotor schema units when a stimulus card is presented
$w_{i,j}^{rule}$	0.40	Weight values from cognitive to sensorimotor schema
$w_{neg}$	0.00	Negative Reward Sensitivity
$m_{r}$	0.00	Memory for negative feedback
$w_{stn}$	1.20	Weight from cortex to subthalamic nucleus
$w_{str,gpi}$	−1.00	Weight from striatum to globus pallidus (internal segment)
$w_{stn,gpe}$	−1.00	Weight from subthalamic nucleus to globus pallidus (external segment)
$w_{stn,gpi}$	0.90	Weight from subthalamic nucleus to globus pallidus (internal segment)
$w_{stn,gpe}$	0.90	Weight from cortex to globus pallidus (external segment)
$w_{gpe,gpi}$	−0.30	Weight from globus pallidus (external) to globus pallidus (internal)
$\varepsilon_{str}$	0.40	Striatum learning rate
$\varepsilon_{sma}$	0.50	Sensorimotor schema learning rate
$\theta_{A}$	$N\left(4000,400^{2}\right)$	Schema integration selection threshold (normally distributed)
$\theta_{s}$	0.50	Static schema selection threshold
$\alpha_{sma}$	8.00	Activation gain of SMA units
$\alpha_{pfc}$	8.00	Activation gain of PFC units
$\alpha_{stn}$	8.00	Activation gain of Subthalamic Nucleus units
$\alpha_{gpi}$	8.00	Activation gain Globus Pallidus (int.) units
$\alpha_{gpe}$	8.00	Activation gain of Globus Pallidus (ext.) units
$\alpha_{thal}$	8.00	Activation gain of Thalamus units
$\alpha_{str,pfc}$	8.50	Activation gain of striatal units for cognitive schemas
$\alpha_{str,sma}$	8.50	Activation gain of striatal units for sensorimotor schemas
$\beta_{thal}$	0.45	Activation threshold of Thalamus units
$\beta_{sma}$	0.40	Activation threshold of SMA units
$\beta_{pfc}$	0.50	Activation threshold of PFC units
$\beta_{str,pfc}$	0.50	Activation threshold of striatal units for the PFC
$\beta_{str,sma}$	0.50	Activation threshold of striatal units for the sensorimotor schemas
$\beta_{stn,pfc}$	0.30	Activation threshold of subthalamic units for the PFC
$\beta_{gpe,pfc}$	0.25	Activation threshold of globus pallidus (ext) units for the PFC
$\beta_{gpi,pfc}$	0.25	Activation threshold of globus pallidus (int) units for the PFC
$\beta_{str,sma}$	0.50	Activation threshold of striatal units for the SMA
$\beta_{stn,sma}$	0.30	Activation threshold of subthalamic units for the SMA
$\beta_{gpe,sma}$	0.25	Activation threshold of globus pallidus (ext) units for the SMA
$\beta_{gpi,sma}$	0.25	Activation threshold of globus pallidus (int) units for the SMA
$\zeta_{stim}$	U(−0.2,+0.2)	Uniformly distributed noise added to external stimulus $o_{stim}$
$\zeta_{str,i}$	U(−0.1,+0.1)	Uniformly distributed noise in $\beta_{str}$ calculation
$\zeta_{sma}$	U(−0.1,+0.1)	Uniformly distributed noise in $\alpha_{sma}$ calculation

Most parameters are self explanatory, but further comment is required on some. Firstly, the noise parameters ensure variability or stochasticity in the model's behaviour, while the variable schema integration threshold introduces variance into the model's response time. Secondly, following, e.g., Amos (2000), we associate $\beta_{str}$, the saturation threshold of the striatum, with the concentration of striatal dopamine, and hypothesise that $\varepsilon_{str}$, which governs adaptation of $\beta_{str}$ (see Equation (10)), is compromised in PD. Thirdly, we assume that cortical learning involves adaptation of the slope of the saturation function of cortical schemas (since decreased slope lessens sensitivity and provides more opportunity for competition). This is governed by the parameter $\varepsilon_{sma}$. Finally, as noted above (Equation (12)), $w_{neg}$ determines the model's sensitivity to negative reward, i.e., the degree to which feedback influences adaptation of $\beta_{str}$.

### 3.5 Operation of the model

When a card is presented its features activate the respective sensorimotor schemas by the quantity $o_{stim}$. For instance, two red crosses activate the first, the second, and the third schema, but not the fourth (four blue circles) because there is no common feature (see Fig. 1). In the meantime, the top-down constant excitation $o_{ext}$ feeds the cognitive units. Once the cognitive schemas are activated they pass activity down to the sensorimotor schemas according to the selected schema rule. The signal is scaled by $w_{rule}$, and added to the previous values gathered from the stimuli, and then integrated over time until a selection can be made, in the same manner as the higher level nodes. When cognitive schemas are not strong enough to influence motor schemas, action selection may be driven by stimulus features only. This basic model is complemented by a mechanism that resolves competition between schemas within each hierarchical level: cognitive and sensorimotor schema nodes feed into two parallel computational mechanisms that simulate basal ganglia functions and each returns a signal in the form of inhibition to the individual channels at each level (Fig. 3).

## 4 Simulation study 1: basic model performance

With appropriate settings of parameters (see Table 1), the model as described above is able to complete the WCST with few errors and high sorting accuracy. Thus, Table 2 shows standard descriptive statistics (for cards correctly sorted, categories achieved and errors of each type) based on 100 simulated runs when the parameters are set to their default values (i.e., the values in Table 1). Studies including ambiguous cards and longer standard run lengths (e.g. Stuss et al., 2000) typically report healthy control participants correctly sorting about 50 cards out of 64 cards, while achieving on average 4 categories and producing 7 perseverative errors and 1 set loss error. With the default parameter values, the model does slightly better than this, but direct comparison of behavioural measures with previous empirical work is not possible because of procedural differences in administration of the task. In particular, in the studies of Barceló (2003) and Lange, Seer, et al. (2016), which used unambiguous cards (as used here and as required to identify integration errors), rule changes occurred very frequently (for example, the feedback “shift” appears after a random number of correct sorts with a median value of 3.5 trials in the study of Lange, Seer, et al., 2016) rather than after 10 consecutive correct sorts. Such frequent shifts severely limits the number of opportunities for set loss errors. Moreover previous work has also shown that more frequent rule changes increase the likelihood of perseverative errors (Grant and Berg, 1948).

**Table 2:** Mean (s.d.) of dependent measures for simulation study 1, based on 100 simulation runs, each with default parameter values (as given in Table 1) and including 64 unambiguous cards.

Measure	Value
Card correctly sorted	54.38 (1.85)
Categories achieved	4.87 (0.37)
Perseverative Errors	5.39 (0.85)
Set Loss errors	0.34 (0.62)
Integration Errors	0.02 (0.14)
RT (post positive feedback, in cycles)	129.10 (1.14)
RT (post negative feedback, in cycles)	144.01 (6.47)

Table 2 also shows the mean (and standard deviation) response time (in processing cycles) following positive and negative feedback (i.e., on trials after a correct response, and on the trials following an error). Like the healthy controls of Lange, Seer, et al. (2016), the model takes longer to respond on trials following an error (where a rule change is required) than on trials following positive feedback.

**Fig. 5:**
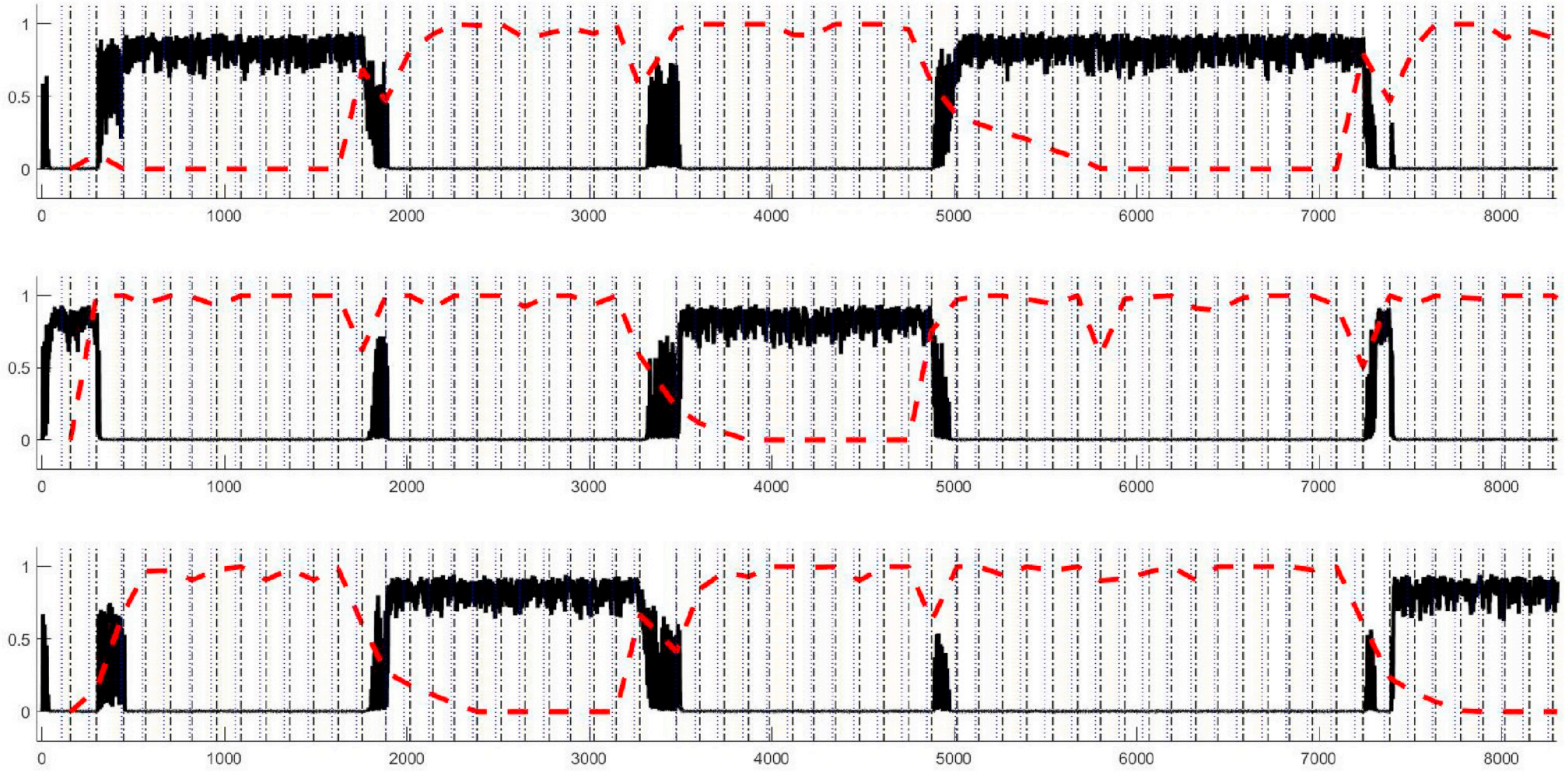
Extract from a typical simulation run. The top plot represents the *sort by colour* schema, the middle one represents the *sort by shape* schema and the bottom one is the *sort by number* schema. The thick black line represents the schema activation value. The dashed red line represents the value of $\beta_{str}$. The black dashed vertical line indicates a new trial and the activation of sensorimotor schemas (and therefore a response). (For interpretation of the references to colour in this figure legend, the reader is referred to the Web version of this article.)

**Fig. 6:**
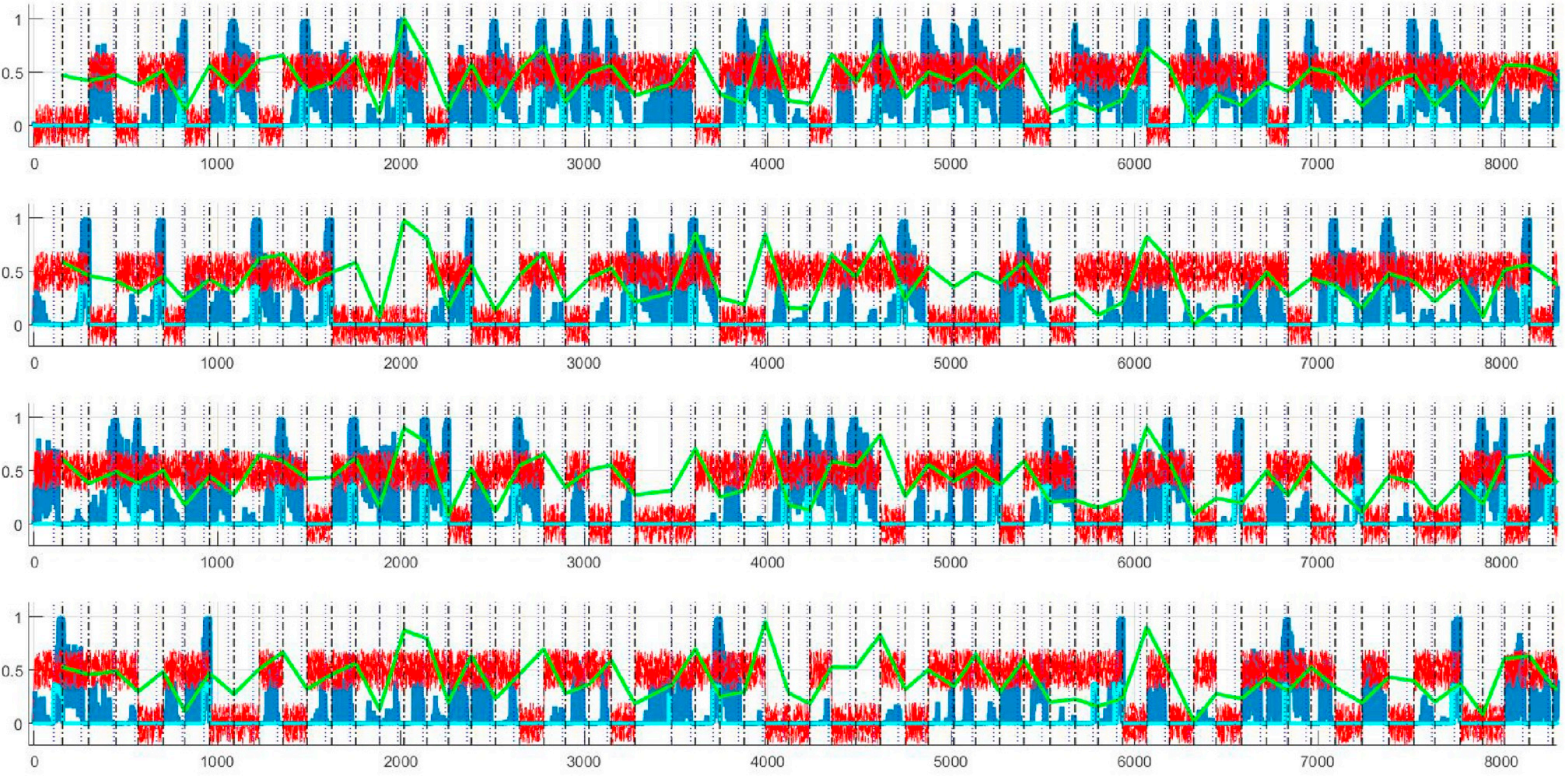
Extract from a typical simulation run. The four plots represent the four sensorimotor schemas. The dashed red line represents the value of $o_{stim}$, that is the fact a stimulus card has a feature in common with that target. The dark blue line indicates the activation of the cognitive schemas, while the light blue line is the top-down input resulting from multiplying that by the relevant weights. The green line is the minmax normalised value of $\alpha_{sma}$. As in Fig. 5, trials are indicated by the black dashed vertical line. (For interpretation of the references to colour in this figure legend, the reader is referred to the Web version of this article.)

The activations of cognitive schema nodes and sensorimotor schema nodes for a part of one instance of a model run with default parameter settings are shown in Fig. 5, Fig. 6 respectively. As can be seen from Fig. 6 (red line) the model selects one motor response following presentation of each card. As can be seen from Fig. 5, the model establishes one sorting schema (e.g., sort by colour) following the first trial. This remains active until negative feedback is received after 10 correct trials (around cycle 1900). Following this, there is a period of exploration before the new sorting schema (sort by number) is determined, and then applied on successive trials. The basic model is therefore able to use initial feedback to correctly determine and activate the appropriate cognitive level schema and to switch to a new sorting schema following negative feedback.

## 5 Simulation study 2: modelling Parkinson's disease

### 5.1 Rationale

As noted above, we hypothesis that $\varepsilon_{str}$ which determines sensitivity of learning within the basal ganglia system to reward prediction error (see Equation (10)), and which subsequently affects the gain of striatal units, is compromised in Parkinson's disease. In order to model PD we therefore reduce $\varepsilon_{str}$ from its default of 0.4. However, PD is not a homogenous disorder and other aspects of basal ganglia adaptation are likely to be disrupted in the disorder. Two possibilities (from Equation (12)) are sensitivity to reward ($w_{neg}$) and the extent to which the calculation of feedback is sensitive to feedback from previous trials ($m_{r}$). In modelling PD we therefore consider four scenarios in comparison to the default parameter settings: reduced $\varepsilon_{str}$; reduced $\varepsilon_{str}$ with increased $w_{neg}$; reduced $\varepsilon_{str}$ with increased $m_{r}$; and reduced $\varepsilon_{str}$ with both increased $w_{neg}$ and increased $m_{r}$.

### 5.2 Method

Four virtual participant groups, PD_1_ to PD_4_, were defined by altering the key parameters from their default values as described above. Table 3 shows the values of the three critical parameters for each group. 100 simulations were then run for each group and dependent measures (corresponding to those in Table 2 for the healthy control simulations) were calculated.

**Table 3:** Parameter values for each virtual participant group in simulation study 2.

Parameter	PD_1_	PD_2_	PD_3_	PD_4_
$\varepsilon_{str}$	0.10	0.10	0.10	0.10
$w_{neg}$	0.00	0.65	0.00	0.65
$m_{r}$	0.00	0.00	0.60	0.60

### 5.3 Results

Table 4 shows the mean (and standard deviations) for all dependent measures and for each simulated patient group. For the most part, the four groups show similar profiles in relation to the data from simulation study 1 (i.e., simulated healthy control participants). Thus, in all cases fewer cards are sorted correctly and fewer categories are achieved.^3^ With respect to errors, in all cases more perseverative errors and more integration errors are produced than in simulation study 1. However, set loss errors show a different profile. They are elevated with respect to the healthy control simulations only in the PD_2_ group.

**Table 4:** Mean (s.d.) of dependent measures for simulation study 2, based on 100 simulation runs each including 64 unambiguous cards, for each simulated PD group.

Measure	PD_1_	PD_2_	PD_3_	PD_4_
Card correctly sorted	45.57 (3.20)	41.43 (10.71)	43.53 (2.86)	38.18 (9.76)
Categories achieved	3.96 (0.35)	3.07 (1.39)	3.84 (0.39)	2.94 (1.45)
Perseverative Errors	12.33 (1.62)	10.12 (4.59)	13.40 (1.76)	12.81 (4.73)
Set Loss errors	0.36 (0.61)	0.94 (1.23)	0.25 (0.46)	0.47 (0.72)
Integration Errors	0.86 (1.12)	1.13 (1.93)	1.67 (1.56)	1.95 (2.65)
RT (post positive feedback, in cycles)	130.80 (1.87)	138.95 (14.97)	133.00 (2.63)	141.22 (12.41)
RT (post negative feedback, in cycles)	148.16 (6.92)	149.05 (16.10)	154.92 (7.35)	157.60 (15.15)

In comparison to simulation study 1, response times on trials following positive feedback also vary across the groups, being substantially longer in the case of PD_2_ and PD_4_, marginally longer in the case of PD_3_, and similar in the case of PD_1_. As in the healthy control simulations, response times are longer on trials following negative feedback than on trials following positive feedback, but the difference in response times between the trial types varies across the groups, being less than in simulation study 1 for PD_2_, similar in PD_4_, and greater in PD_1_ and PD_3_.

### 5.4 Discussion

Recall that, in comparison to healthy control participants, Parkinson's disease patients sort fewer cards correctly and hence achieve fewer categories. They also tend to produce more perseverative errors and more set loss errors, though the work of Lange et al. (2017) suggests that they do not produce more integration errors. At the same time, PD patients are slower at sorting cards than healthy controls, and this slowing is independent of feedback. That is, the slowing following positive feedback is similar in magnitude to that following negative feedback.

These qualitative findings are largely, though not completely, replicated by the four groups considered here. All groups show fewer cards correctly sorted and fewer categories achieved than in simulation study 1. Equally, all groups show elevated rates of perseveration, but in contrast to the patient data, groups PD_1_ and PD_3_ do not show elevated rates of set loss errors (and the increase in these errors for group PD_4_ is relatively small). Also in contrast to the patient data, all groups show elevated levels of integration errors in comparison to simulation study 1, however this may be because such errors were almost completely absent in that simulation study. With respect to response time, only groups PD_2_ and PD_4_ show a substantial lengthening in comparison to simulation study 1, with the effect of negative feedback being smaller for PD_2_ than in simulation study 1 (10.10 cycles versus 14.91 cycles), but similar for PD_4_ (16.38 cycles).

To summarise, based purely on the simulated dependent measures reported here, groups PD_2_ and PD_4_ probably provide the most empirically adequate accounts of PD patient behaviour, suggesting that PD is plausibly accounted for within the model by reduced $\varepsilon_{str}$ (equivalent to reduced sensitivity to striatal dopamine) and increased $w_{neg}$ (equivalent to increased sensitivity to negative reward). That is, the simulated data suggest that PD is better modelled by variation of multiple parameters than by variation of a single parameter.

As an aside, these simulations also suggest that within the model the tendencies towards perseveration and set loss can dissociate, in that groups PD_1_ and PD_3_ show elevated perseverative errors in the absence of elevated set loss errors. Arguably, the two types of error have different sources, with perseverative errors reflecting a failure in reactive control (i.e., following negative feedback) and set loss errors reflecting a failure in proactive control (and in particular in task set maintenance). Indeed, some studies of WCST and healthy aging have shown dissociations between the two types of error (e.g., Caso & Cooper, in preparation; Paolo et al., 1996). This is consistent with arguments such as those of Rhodes (2004), who suggested on the basis of a meta-review that age-related perseveration is moderated by the number of years of education, with more educated participants tending to commit fewer perseverative errors.

Note that in the simulations studies presented thus far, each group is simulated by a single set of parameter values. This is tantamount to considering each group to consist of a number of identical individuals. This is an implausible assumption, though it is helpful in determining the central tendencies of dependent measures in each group. Clearly individuals within a group are likely to differ in the settings of the various parameters. Furthermore, one parameter not considered in this stimulation study and whose value might possibly be affected by Parkinson's pathology is $\varepsilon_{sma}$, which regulates cortical learning (cf. Equation (4)). This is because dopamine depletion caused by Parkinson's disease may not only impact striatal areas, but also cortical areas that receive dopaminergic projections from the ventral tegmental area in the midbrain, and this could potentially affect both learning and active maintenance of temporary representations (Narayanan et al., 2013; van Schouwenburg et al., 2010). A full exploration of the effect of varying this parameter in conjunction with the other three parameters considered here is provided in the appendix, where it is shown that decreasing $\varepsilon_{sma}$ increases response time, and that low values of $\varepsilon_{sma}$ generally result in more set lose errors than higher values. Given this, and the previous comment about modelling groups of non-identical individuals, in subsequent simulations we assume that healthy control participants are plausibly modelled by a four-dimensional region of parameter space containing the point identified in simulation study 1, and that Parkinson's disease patients are plausibly modelled by a non-overlapping region in which $\varepsilon_{str}$ and $\varepsilon_{sma}$ are reduced and $w_{neg}$ and $m_{r}$ are elevated.

## 6 Simulation study 3: mapping internal processes to ERP components

As seen in simulation study 2, the model reliably shows how the mapping between the stimuli generated by the environment and error frequencies is plausibly altered by neurobiologically grounded parameters. We now turn to examining the internal processes of the architecture, and how they relate to two ERP components, the error-related negativity (ERN) and the posterior switch positivity (PSP), and specifically to how those components are modulated as a consequence of Parkinson's pathology.

### 6.1 Error-related negativity (ERN)

The error-related negativity (ERN) is a brain potential with a frontocentral distribution that peaks approximately 100 ms after a specific event, usually an error committed by participants performing reaction time tasks (Gehring et al., 1993). The ERN is generally believed to be generated by the Anterior Cingulate Cortex (ACC; van Veen and Carter, 2002) but several other brain areas present a signal with the same signature. Brázdil et al. (2002) analysed intracerebral recordings in a simple visual oddball paradigm and showed how an ERN signal may be generated in the rostral ACC as well as the pre-supplementary motor area (pre-SMA), the orbitofrontal cortex (OFC) and, somewhat unusually, the mesiotemporal areas. In addition to the presence of multiple sources for this signal, the latency differences between posterior and anterior components suggested that signals originate from caudal areas and are later processed in frontal regions. Critically for the current work, across a range of tasks the ERN has been found to be attenuated in PD patients (Falkenstein et al., 2001).

In order to be an accurate representation of the ERN, a function of the internal variables of the model must satisfy a number of criteria. First, its value after an error must be greater than when a response is correct (Coles et al., 2001). Secondly, the value of the signal has to drop below baseline immediately before response and then peak after response. Since the model functions in processing cycles rather than real time, and since not all variables are continuous, the relative positions of the signal compared to the response is a meaningful criterion to assess whether the signal is a proxy for the ERN. Finally, if it is to reflect error it must be at least moderately correlated with the conflict values at response. Equation (13) satisfies these requirements.

(13) $$\begin{array}{l} ERN_{corr}\leftarrow -absmax\left(o_{sma}^{t}-o_{sma}^{t-1}\right)\\ ERN_{incorr}\leftarrow -absmax\left(o_{sma}^{t}-o_{sma}^{t-1}\right)\\ ERN\leftarrow ERN_{corr}-ERN_{incorr} \end{array}$$

where $absmax\left(x\right)=x_{\mathrm{argmax}\left|x_{i}\right|}$.

Equation (13) states that a proxy for the ERN signal is obtained by finding the temporal variation for each sensorimotor schema and taking the one with the greatest absolute activation, preserving the sign. ERN attenuation for each correct and incorrect trial is measured as the difference between ERN signals between time-steps. The final ERN signal is then calculated as the (weighted) difference between the ERN in the correct conditions and the ERN in the incorrect condition.

**Fig. 7:**
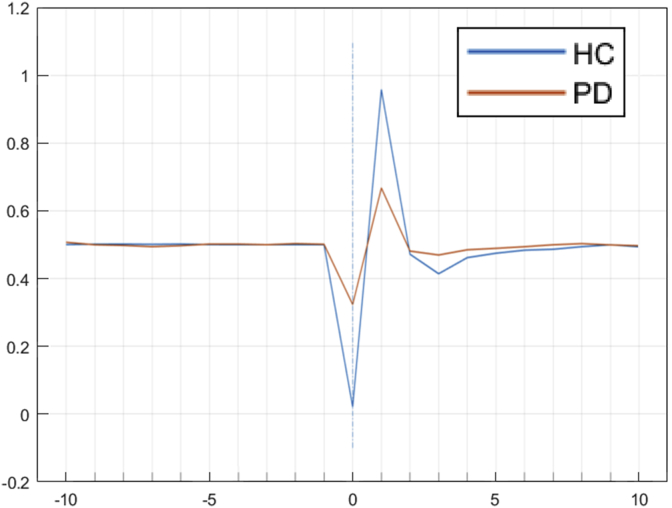
The mean ERN signal (smoothed) for twenty virtual HC individuals and twenty virtual PD individuals. The signal drops below the baseline and then peaks, and it is attenuated for the virtual PD participants.

A simulation of the mean signal for twenty virtual healthy controls (HC) and twenty virtual PD patients is shown in Fig. 7. Here, HC and PD patients are simulated by using the midpoint of each variable in the two parameter spaces (HC and PD), as described in Table 5. Critically, the ERN is attenuated in simulated PD patients, mirroring the clinical findings of Falkenstein et al. (2001) obtained over a range of executive tasks.

**Table 5:** Table of the parameter range for Healthy controls (HC) and Parkinson's individuals (PD). Parameters have been evaluated in 4 equally spaced subintervals in the specified range, for a total of 256 (=4×4×4×4) datapoints for each group.

	$\varepsilon_{str}$	$\varepsilon_{sma}$	$w_{neg}$	$m_{r}$
HC	0.40 – 0.70	0.50−0.70	0.00−0.20	0.00−0.20
PD	0.05 – 0.20	0.30−0.50	0.50−0.80	0.50−0.70

### 6.2 Posterior switch positivity (PSP)

Empirically, the Posterior Switch Positivity (PSP) is calculated as the difference between the sustained peaks from 600 ms to 800 ms (Sustained Parietal Positivity; SPP) produced by shift and repeat trials in the posterior parietal area (Lange et al., 2017). This neural activity is widely believed to reflect set-shifting processes between cognitive sets (Karayanidis et al., 2010). In order to be an accurate representation of the PSP, a function of the internal variables of the model must produce a signal that is present in the set-shifting trials but attenuated in the subsequent ones. The same *absmax* function previously used for ERN meets these criteria if applied to the activation value of cognitive schemas. PSP is therefore computed for the model by calculating the values of the SPP for both shifting trials and the subsequent ones, as can be seen in Equation (14). PSP attenuation is then measured as the mean difference between the peaks of two signals.

(14) $$\begin{array}{l} {\text{SPP}}_{t}\leftarrow absmax\left(o_{pfc}^{t}-o_{pfc}^{t-1}\right)\\ {\text{PSP}}_{att}\leftarrow {\text{SPP}}_{shift}-{\text{SPP}}_{first\;cue} \end{array}$$

**Fig. 8:**
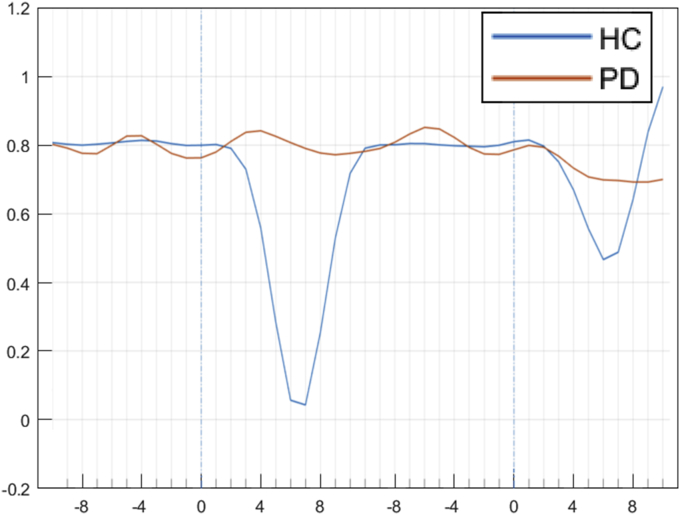
The mean rate of change for set-shifting (left blue dashed line) and subsequent trials (right blue dashed lines), from which PSP is calculated. The signal is again averaged across twenty virtual HC individuals and twenty virtual PD individuals. (For interpretation of the references to colour in this figure legend, the reader is referred to the Web version of this article.)

As shown in Fig. 8, simulated PSP is attenuated in PD participants compared to HC controls.

## 7 Simulation study 4: relations between behavioural measures and ERP amplitudes

Simulation study 4 explores the relationship between the frequency of each error type and signal attenuation, with the aim of comparing and generating predictions for Parkinson's Disease. An accurate comparison of performance with previous empirical work is unobtainable because of procedural differences in administration and scoring of the task, and the fact that the data of Lange, Seer, et al. (2016) are pooled across patients with and without dopaminergic replacement therapy. Thus, in order to identify a parameter space for healthy controls and individuals with PD we supplement the behavioural results of Lange, Seer, et al. (2016) with the theoretical considerations illustrated above to generate a set of non-overlapping parameter spaces corresponding approximately to the two groups, as in Table 5.

Simulations were first run of 10 virtual participants for all possible combinations of the four critical parameters in the first row of Table 5. This set of parameters should be able to simulate a wide range of young healthy participants. Spearman correlation coefficients between ERN and PSP attenuations and frequency of each error were calculated. Results are shown in Table 6.

**Table 6:** Spearman's correlation coefficients between ERN and PSP attenuation signals and the frequencies of the different types of error in a parameter space associated with healthy controls. N = 2560. ** is p<.001, * is p<.05

	PE	SL	IE
${\text{ERN}}_{att}$	−.37**	.12*	.25**
${\text{PSP}}_{att}$	−.31**	.12	.20*

**Fig. 9:**
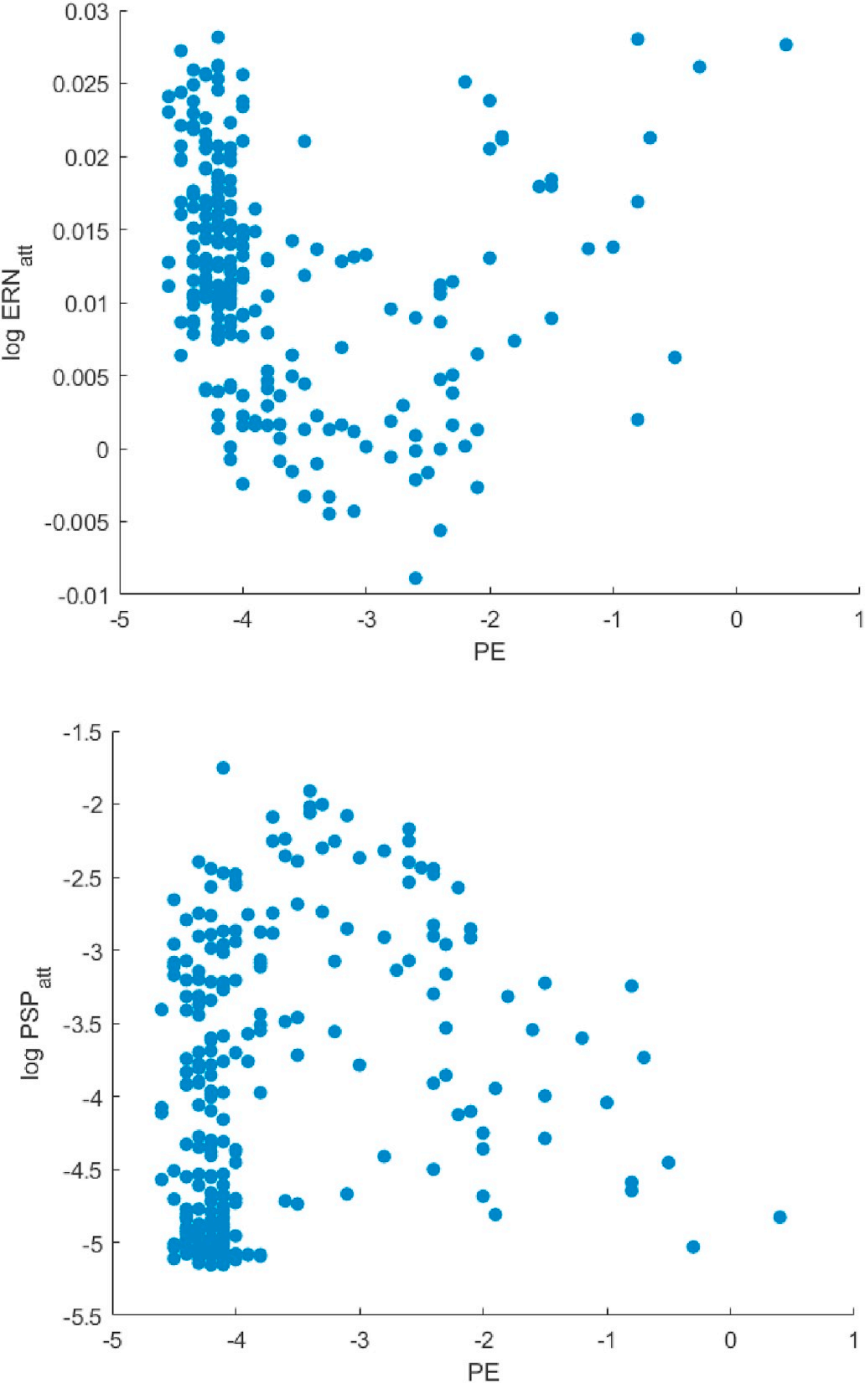
Scatterplot of datapoints in simulated HC (Healthy Controls space). Ordinate axis is logarithmic.

In the simulation of healthy controls, ERN and PSP show similar relations to behavioural measures, and in particular are negatively correlated with PE. However, inspection of the relevant scatter plots reveals a non-monotonic relationship between the variables (Fig. 9), thus limiting interpretation of the values in Table 6. This suggests that the HC space we designed is heterogeneous and meaningful inferences regarding relations between errors and ERPs are not possible.

Simulations of 10 virtual participants were then run for the parameters associated with PD in Table 5. Spearman correlation coefficients between ERN and PSP attenuations and the frequency of each error type were calculated. Results are shown in Table 7.

**Table 7:** Spearman's correlation coefficients between ERN and PSP attenuation signals and the frequencies of the different types of errors in a parameter space associated with PD. N = 2560. ** is p<.001, * is p<.05

	PE	SL	IE
${\text{ERN}}_{att}$	.37**	.38**	−.08
${\text{PSP}}_{att}$	.33**	.10	−.09

**Fig. 10:**
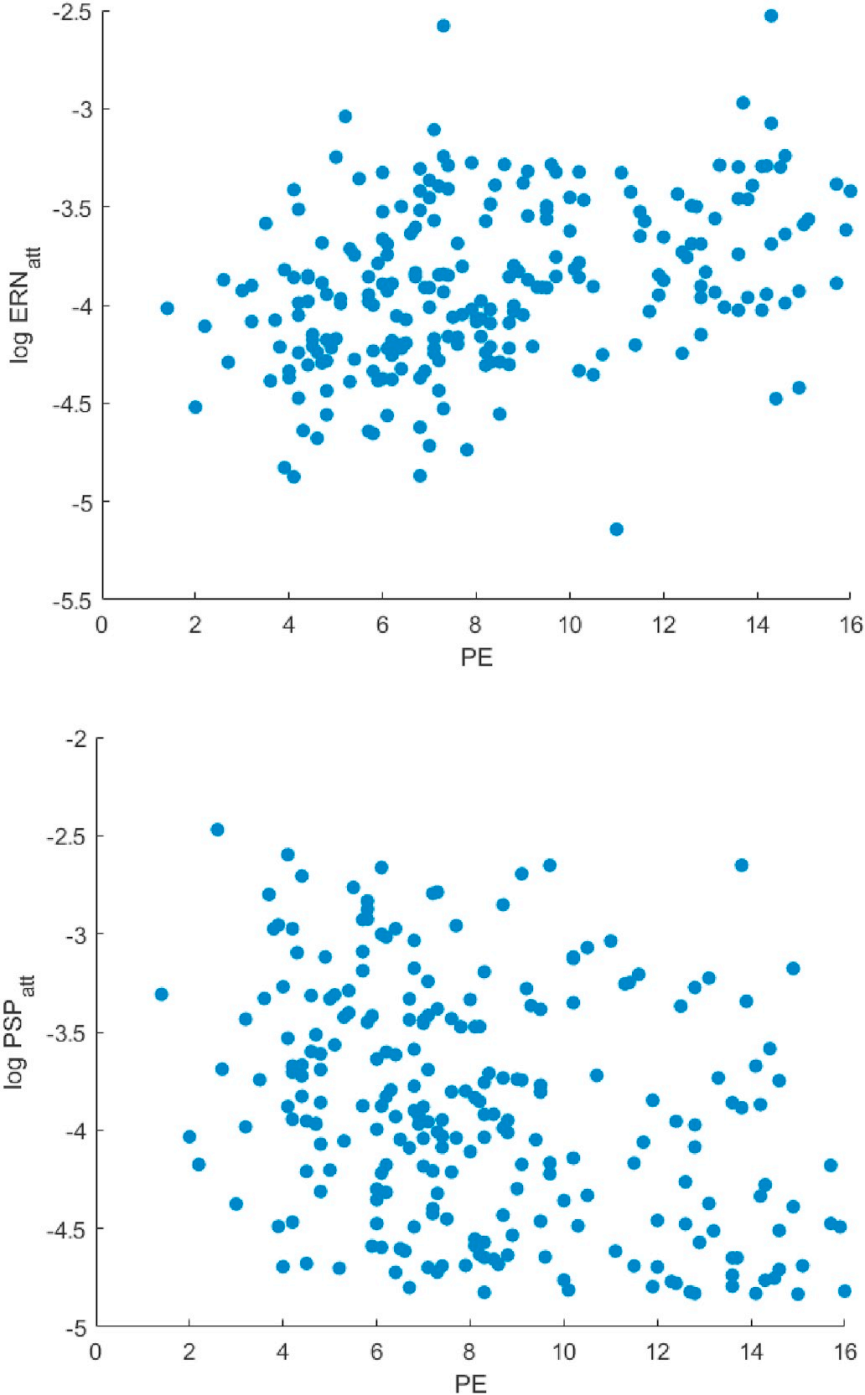
Scatterplot of datapoints in simulated PD (Parkinson's Disease space). Ordinate axis is logarithmic.

In this case, inspection of the scatter plots shows monotonic relationships between variables (Fig. 10), suggesting that the simulated PD group is more homogenous than the simulated HC group and that confident inferences regarding errors and neurophysiological markers is possible. The strong correlation between PE and both the attenuation of ERN and of PSP suggests that both decreased set-shifting and decreased response conflict may be responsible for Perseverative Errors. Since our model implements a response conflict mechanism that generates the ERN signal only in the motor schemas independently of dopamine signal in the midbrain, this is consisted with Holroyd et al. (2002), who differentiate between motor and error-related processes in the context of a Flanker task, and suggest that PD disrupts exclusively the motor process. There is also support for the notion that the PSP signal, which is considered to be a signature for cognitive set-shifting (Lange et al., 2017), is associated with Perseverative Errors, albeit the evidence is limited to a pathological state (PD) and does not apply in the general case.

The difference in correlation between Set Loss errors and ERN versus Set Loss errors and PSP is also of interest. It constitutes an experimental prediction: in participants with PD, the number of SL errors should increase as the ERN signal becomes smaller, but be independent of the size of PSP.

## 8 Simulation study 5: on the relation between model parameters and ERP components

### 8.1 Rationale and method

Simulation study 5 complements simulation study 4 by addressing the relationship between model parameters (rather than simulated behavioural measures) and attenuation of the simulated ERP components. Firstly, 20 simulations were run for each value of $\varepsilon_{str}$ ranging from 0.00 to 1.00 in steps of 0.05 and three values of $\varepsilon_{sma}$ (0.2, 0.5 and 0.8). All other parameters were held at their default values. Attenuation of the ERN and ESP signals was calculated for each point in the parameter space. Secondly, the effect of varying $\varepsilon_{sma}$ (from 0.00 to 1.00 in steps of 0.05) was explored in a similar manner, for three values of $\varepsilon_{str}$ (0.2, 0.5 and 0.8). Finally, the effect of varying $w_{neg}$ (from 0.00 to 1.00 in steps of 0.05) was explored in a similar manner, for the same three values of $\varepsilon_{str}$.

### 8.2 Results and discussion

**Fig. 11:**
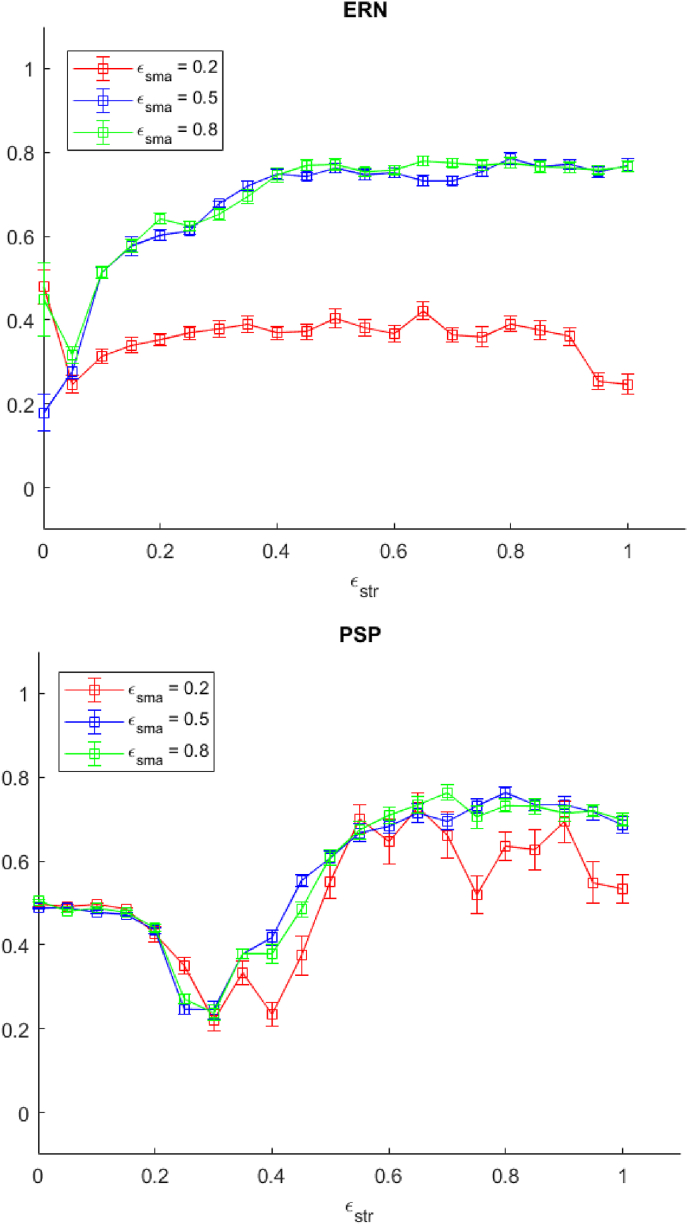
The ERN and PSP attenuation (minmax normalised) as a function of $\varepsilon_{str}$. The red, blue, and green line represent values of $\varepsilon_{str}$ for 0.2, 0.5, 0.8, respectively. Error bars show one standard error from the mean. (For interpretation of the references to colour in this figure legend, the reader is referred to the Web version of this article.)

As discussed earlier, reducing $\varepsilon_{str}$ is hypothesised to correspond to a reduction of dopamine concentration in the basal ganglia circuits, as seen in PD pathology. As shown in Fig. 11, reduction in $\varepsilon_{str}$ results in attenuation of the simulated ERN, This supports the reinforcement learning model of the ERN in which this neural activity is a signature of prediction error generated by midbrain dopamine neurons and relayed to the prefrontal cortex (Holroyd and Coles, 2002). It is also compatible with ERN attenuation in PD (Beste et al., 2009). With regard to the PSP, attenuation of the signal following $\varepsilon_{str}$ reduction is consistent with what has been observed in our model, though it runs against the view that PSP is a product of cortico-cortical computation only (Karayanidis et al., 2010).

**Fig. 12:**
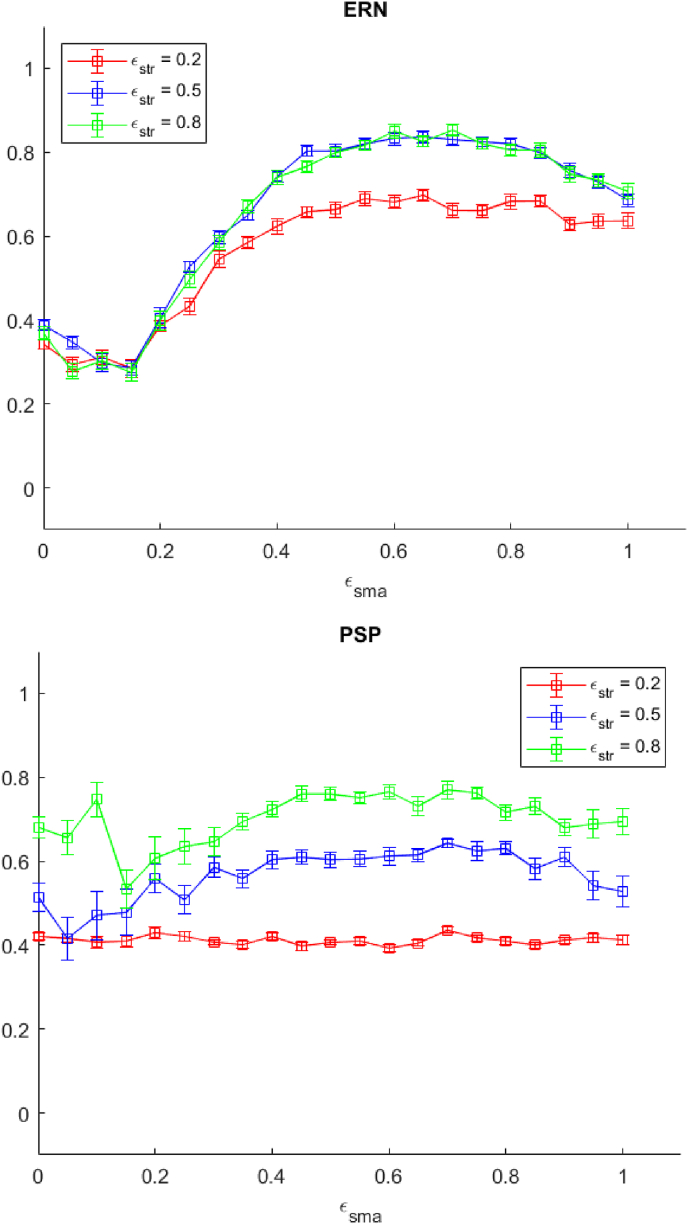
The ERN and PSP attenuation (minmax normalised) as a function of $\varepsilon_{sma}$. The red, blue, and green line represent values of $\varepsilon_{str}$ for 0.2, 0.5, 0.8, respectively. Error bars show one standard error from the mean. (For interpretation of the references to colour in this figure legend, the reader is referred to the Web version of this article.)

A suboptimal value of $\varepsilon_{sma}$, which we regarded as an indicator of deterioration of cognitive control, has an effect on ERN attenuation but not on the PSP (see Fig. 12). Despite an increase in Set Loss errors for high values of $\varepsilon_{sma}$, ERN attenuation is unaffected in that range. On this account, this ERP profile may potentially identify prodromal executive dysfunctions in PD. These have been clinically recognised but research has found them difficult to pinpoint (Fengler et al., 2017).

**Fig. 13:**
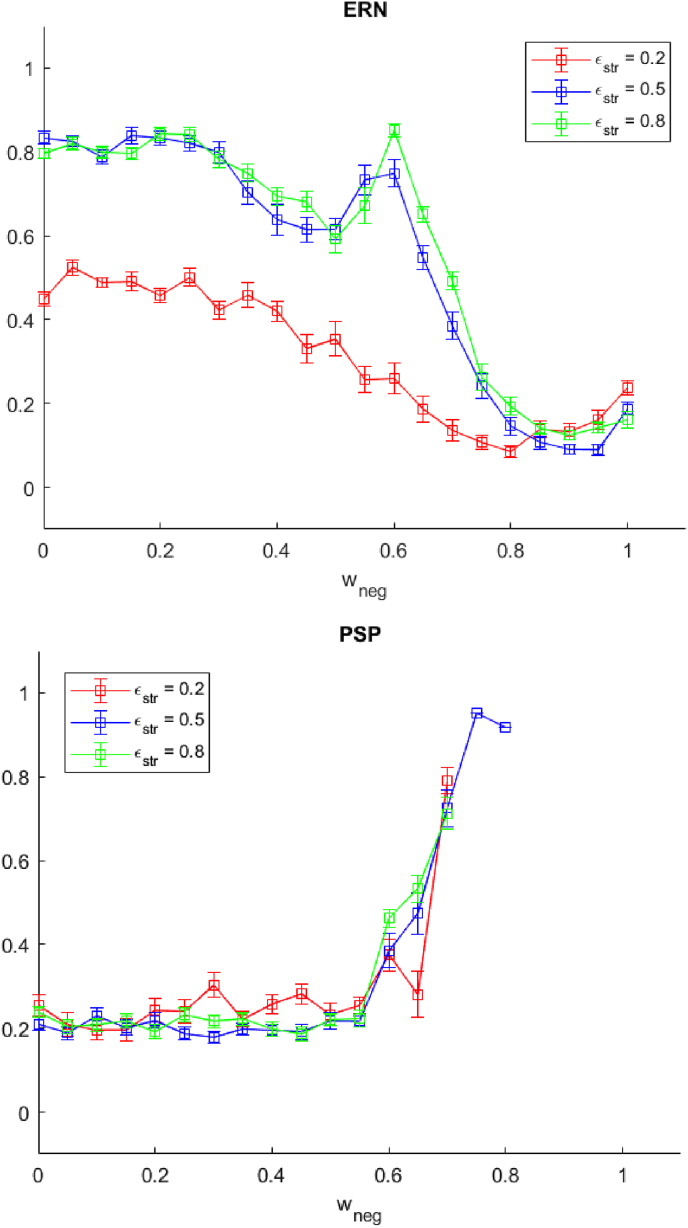
The ERN and PSP attenuation (minmax normalised) as a function of $w_{neg}$. The red, blue, and green line represent values of $\varepsilon_{str}$ for 0.2, 0.5, 0.8, respectively. Error bars show one standard error from the mean. When the value of $w_{neg}$ becomes too high, many PE are generated and the model does not switch between schemas. Consequently the PSP cannot be calculated. (For interpretation of the references to colour in this figure legend, the reader is referred to the Web version of this article.)

Manipulation of reward sensitivity ($w_{neg}$) also yields interesting results, in that it makes counter-intuitive and opposite predictions regarding ERP and PSP attenuations (see Fig. 13). From the clinical standpoint, reduction in reward sensitivity is generally believed to contribute to apathy, defined as a lack of motivation for goal-driven behaviour (Muhammed et al., 2016), and it is present in at least one third of Parkinson's Disease patients. Apathy appears to be unrelated to disease progression, personality traits, or depression (Pluck and Brown, 2002). The neural substrate of apathy is unclear, but dopamine is not the only neurotransmitter involved in this disorder (Dujardin et al., 2007). One possibility suggested by our results is that the presence of a dissociation in ERN and PSP attenuation in the WCST may constitute a potential biomarker for diminished reward sensitivity, and hence apathy. A caveat is that the parametrisation we introduced directly affects negative reward sensitivity, and not positive reward sensitivity, although these are known to be dissociated, even at the level of ERN signals (Boksem et al., 2008).

## 9 General discussion

### 9.1 Summary of findings

This work constitutes an important step in the development of a computational theory of the cognitive control of schemas. Here we focused not on how these knowledge structures are formed or updated by experience, but on the way they are controlled to produce flexible behaviour. In order to illustrate these processes we produced an activation-based model of the Wisconsin Card Sorting Test consisting of three higher-order schemas representing the application of sorting rules and four lower-order schemas representing actions. We supplemented this architecture with sets of basal ganglia units that resolve the competition between schemas at each level, enabling the evaluation of suboptimal performance of the basal ganglia component when dopamine is depleted, as in the case of Parkinson's Disease. We then examined how the internal variables of the model relate to two ERP components: the error-related negativity (ERN) and the posterior switch positivity (PSP). Finally, we showed that parameterisation distinguishes between perseveration and conflict, and produces distinct ERP signatures for distinct components of Parkinson's Disease dysfunction.

### 9.2 Contention scheduling and the supervisory system revisited

In the original model of contention scheduling presented by Cooper and Shallice (2000) it was argued that, at the computational level, competition between schemas was determined by lateral inhibition, and the strength of lateral inhibition was inversely related to striatal dopamine concentration. This was held to account for the slowing of action initiation in PD patients, but other cognitive deficits associated with PD were not considered. The account offered here of the PD deficit is far more detailed, both in providing more explicit bridging assumptions between the neural and computational levels and in providing an account not just of the slowing of schema selection in PD but also of other cognitive deficits associated with PD, such as increased tendencies toward perseveration and impairment in set maintenance.

Perseveration is accounted for with an implementation of basal ganglia activity that contributes to making the contention scheduling mechanism more neurally grounded. Impairment in set maintenance due to a diminished ability to handle conflict at the level of sensorimotor schemas is instead a feature that could be ascribed to the supervisory system, which biases schemas in a domain-general fashion, irrespective of the nature of the representation itself. The idea that these biasing processes may act at different levels of hierarchical organisation might also constitute a possible account of metacognitive skills (Nelson and Narens, 1990), a view also supported by Fernandez-Duque et al. (2000), who argue on the basis of imaging studies that there is a high degree of overlap between the brain regions supporting metacognitive skills and those involved in conflict resolution and error correction.

The model may also help to better localise some of the hypothesised operations of contention scheduling and the supervisory system. Previous work, based on studies of Ideational Apraxic patients with left temporoparietal lesions (De Renzi and Lucchelli, 1988) and imaging studies of neurologically healthy participants pantomiming action related tasks (Rumiati et al., 2004), has suggested that the associations by which the representations of objects trigger schemas are localised in left temporoparietal regions, while the deficits of frontal patients have been modelled with noise in the schema network (Cooper et al., 2005), suggesting that schemas, or their activations, are maintained in frontal regions. We can, however, be more specific given the theoretical framework of Badre and D'Esposito (2009), that posits a rostro-caudal gradient of abstraction (with rostral/pre-frontal areas supporting activity directed towards more abstract, temporally-extended goals and caudal/premotor areas supporting activity directed towards more immediate, concrete goals). This framework, together with the model, implies that the lower and higher schemas are associated with the premotor (BA 6) and the DLPFC (BA 9, 46), respectively. The use here of a conflict signal for cortical learning (Equation (4)) is novel. Imaging and ERP work (e.g., van Veen and Carter, 2002, as cited above) suggests that this might be mapped onto the Anterior Cingulate Gyrus and pre-SMA, for higher and lower-level conflict respectively. Finally, the model suggests that the basal ganglia units connected to the lower and higher schemas are mapped onto the sensorimotor and associative striatum, respectively.

### 9.3 The basal ganglia and competition resolution

Competition between schemas within our model is effected by the model's basal ganglia component. In contrast to the original model of Cooper and Shallice (2000), this does not require a set of weights that grows with the square of the number of schemas. The mechanism of our model is arguably more energetically efficient and evolutionary plausible (Redgrave et al., 1999). Moreover, each set of basal ganglia units has the mathematical property of instantiating the multi-hypothesis sequential probability ratio test (MHSPR), a test that guarantees an optimal solution for action selection in the presence of noisy stimuli (Bogacz and Gurney, 2007). In addition to the above, it is well established neuroanatomically that corticobasal loops are mostly segregated (Alexander et al., 1986), and that a gradient exists between sensorimotor cortex and association cortex projections to dorsolateral and dorsomedial striatum, respectively (Yin and Knowlton, 2006). This conceptual framework is present in the model, through the independence of information processed in the basal ganglia units at the two different levels.

The idea of the basal ganglia operating in segregated corticothalamic loops has been widely discussed in the literature, and several computational models have been produced. For instance, the seminal work of O'Reilly and Frank (2006) describes a dynamic gating system that controls working memory updating. That work makes a clear distinction between the type of computation performed by prefrontal structures and the basal ganglia. The authors put forward a set of functional demands under which working memory needs to operate in order to accomplish a simple sequential working memory task. However, that model is not directly compatible with schema theory, due to its use of acquired distributed representations (which are themselves learned via contrastive Hebbian learning) rather than explicit schemas. The work by Gurney et al. (2001) is instead unique in that the computational (Redgrave et al., 1999), algorithmic (Bogacz and Gurney, 2007), and implementational (Humphries et al., 2006) levels are kept distinct, while being connected by specific bridge laws. The computational level draws on both evolutionary neuroscience and cybernetics, with the basal ganglia model having been successfully embedded in an embodied robot architecture that processes differently salient sensory and motivational states in a foraging task (Prescott et al., 2006). The algorithmic level uses a population-level signal-processing approach, while the implementational level uses available neurophysiological data from spiking neurons for each population present in the algorithmic level (Humphries et al., 2006) and has recently been shown to be consistent with the internal computations of at least some basal ganglia nuclei (notably the GPe; Suryanarayana, Kotaleski, Grillner and Gurney, 2019). The hierarchical structure of the Cooper and Shallice (2000) model and the computational capabilities of the Gurney et al. (2001) model dovetail when the signal from a schema is conceptualised as channel salience, and further justifies the choice of this action selection model for the arbitration of schemas. It is important to notice that the need for a schema arbitration system does not supersede the need for the supervisory system as defined in Shallice and Burgess (1993). The top-down bias coming from this system is the result of temporary schemas that are created on the basis of overarching (and potentially novel) goals, whilst the basal ganglia selection mechanism acts on pre-existing schemas, and is dependent on the history of rewards.

### 9.4 The ERN, error detection and conflict monitoring

A key feature of our approach is the linking of the operation of the simulated basal ganglia and neural signals corresponding to ERPs through explicit bridging assumptions (Equation (13) and Equation (14)). One such signal is the ERN. Soon after its discovery, the main theory characterising the functional meaning of ERN was the error detection theory (Falkenstein et al., 1989), according to which the brain produces an estimate or prediction of the output, compares it with the response motor signal, and acts on the mismatch by either inducing another motor command or by inhibiting the incorrect motor command. This theory has been argued to be computationally implausible and unable to account for instances where the ERN appears in absence of errors (Yeung et al., 2004).

Our approach is guided by the conflict theory of ERN, but there are several important differences between previous models and the approach adopted here. Our model is not a feedforward neural network architecture (as is the model of Yeung et al., 2004), and nor is it trained by means of changing its weights (as in the model of O'Reilly and Frank, 2006). Rather, it is a signal-based model that assumes a pre-existing hierarchical structure. Similarly to Yeung et al. (2004), we implement both the conflict detection and the regulative role at the level of the response units (recall Equation (4) for its implementation). However, in the neural network model conflict monitoring input is computed with the response unit values, and the output then affects the task representation units. In our model each conflict is handled at the same level of the hierarchy. This is consistent with the possible presence of ERN signals at multiple locations in the brain, where conflict evaluation and subsequent regulations are carried out at the same level of abstraction. Another difference between the two models is the presence of the simulated basal ganglia as an arbitration device. Adding this structure to models of cognitive control has been shown to be important in constraining action selection mechanisms. Stafford and Gurney (2007), for example, demonstrated that a widely accepted model of the Stroop task (the model of Cohen et al., 1990) could not account for effects related to stimulus onset asynchrony (where the onset of the colour and text of the stimulus was not simultaneous), but that this inadequacy of the model could be addressed by the addition of a simulated basal ganglia.

Another key feature of the model is that the selection mechanism at the level of the basal ganglia functions independently of the mechanisms that support cognitive control. In order to demonstrate this, we ran an additional simulation and calculated the maximum Spearman's correlation coefficient (across schemas) between the value of $o_{gpi}$ at each time unit and the value of $o_{sma}$ after an interval of between 1 and 10 cycles. If cognitive control and action selection are independent processes this correlation should be independent of $\varepsilon_{sma}$. This was indeed found to be the case, with the correlation coefficient hovering between −0.55 and −0.60 across the entire range of $\varepsilon_{sma}$ (i.e., from 0 to 1), indicating a near constant relation of moderate/strong magnitude, and suggesting that the selection process remains robust in the absence of cognitive control.

### 9.5 Limitations and future research

One limitation of the present work, which arises from the continuous nature of the WCST, is that in the ERP signal stimulus-locked and response-locked components overlap, with the next stimulus being presented as soon feedback is given. This makes it hard to tease apart stimulus-related and response-related processes. The model also contains several continuous variables, though some values are updated in a discrete fashion. For this reason the shape of the ERP components is not as smooth as one might expect. In fact, the signals obtained from the functions of the internal processing variables should be viewed as proxies for the ERP signals, rather than as precise predictors. Thus they are intended to preserve their main properties, such as differences with respect to the baseline value and temporal relationship among each other, but not the detailed ERP signal. This limitation could be overcome in future research by producing a lower-level computational model where neuronal firing rate is proportional to the activation of schemas and therefore the ERP components preserve their higher-order properties while also displaying a more complex lower-level behaviour.

The use of discrete approximations to continuous variables throughout the model also has some unfortunate consequences. Most critically, for some values of the model's parameters it can cause the cortico-thalamic loop to oscillate, sometimes unpredictably. While this is a problem related to the nature of model implementation, it needs to be resolved in order to fully legitimise the union between schema theory and cognitive neurophysiology through a computational lens.

There are three concomitant priorities for future research: firstly, adapting the model to include more continuous variables so as to produce smoother local signals; secondly, developing a more accurate quantitative model that compares different groups within the PD cohort, in order to distinguish the extent of executive and even emotional dysfunctions; and thirdly, applying the model with a similar parametrisation to other tasks for the purpose of dissociating domain-specific and domain-general mechanisms.

### 9.6 Conclusion

We have shown how an existing cognitive model of schema selection can be elaborated with a neurobiologically plausible model of the basal ganglia, with the combined model including parallel cortico-subcortical loops (one per schema) and the basal ganglia component serving to select between schemas by disinhibiting one of a set of competing loops. The full model is presented within the context of the WCST, though its mechanisms are general. The basal ganglia component includes parameters held to reflect striatal dopamine concentration (specifically $\varepsilon_{str}$), and reduction in the value of this parameter results in the full model showing the characteristics of Parkinson's Disease patients on the WCST (e.g., slower responses and an increased tendency towards perseveration). Moreover variation of other parameters of the model results in the generation of other types of errors (notably set loss errors and integration errors). Finally, we argued that signals within the model may be related to ERP components, and considered two such components: the ERN and the PSP. We then demonstrated how reduction in the value of the $\varepsilon_{str}$ results in attenuation of ERN and PSP signals, as has been found with PD patients. The work therefore demonstrates how cognitive models may make contact with neural-level data (ERPs), and provides both strong support for the Gurney et al. (2001) model of the basal ganglia and a mechanistic account for how the commonly hypothesised relation between striatal dopamine depletion in PD may result in response slowing, perseverative tendencies, and attenuated ERP signals.

## Authors' note

We are grateful to Florian Lange for kindly sharing the anonymised raw data from his work on the Wisconsin Card Sorting Task with PD patients and age-matched healthy controls, and to Alexander Steinke and four anonymous reviewers for their constructive comments. Andrea Caso was supported by an Institutional Strategic Support Fund grant from the Wellcome Trust and Birkbeck, University of London (award reference: 204770/Z/16/Z). The code was implemented in Matlab™ 2019a and is available at http://github.com/AndreaCaso/wcst-pd.

## Figures and Tables

**Fig. 14:**
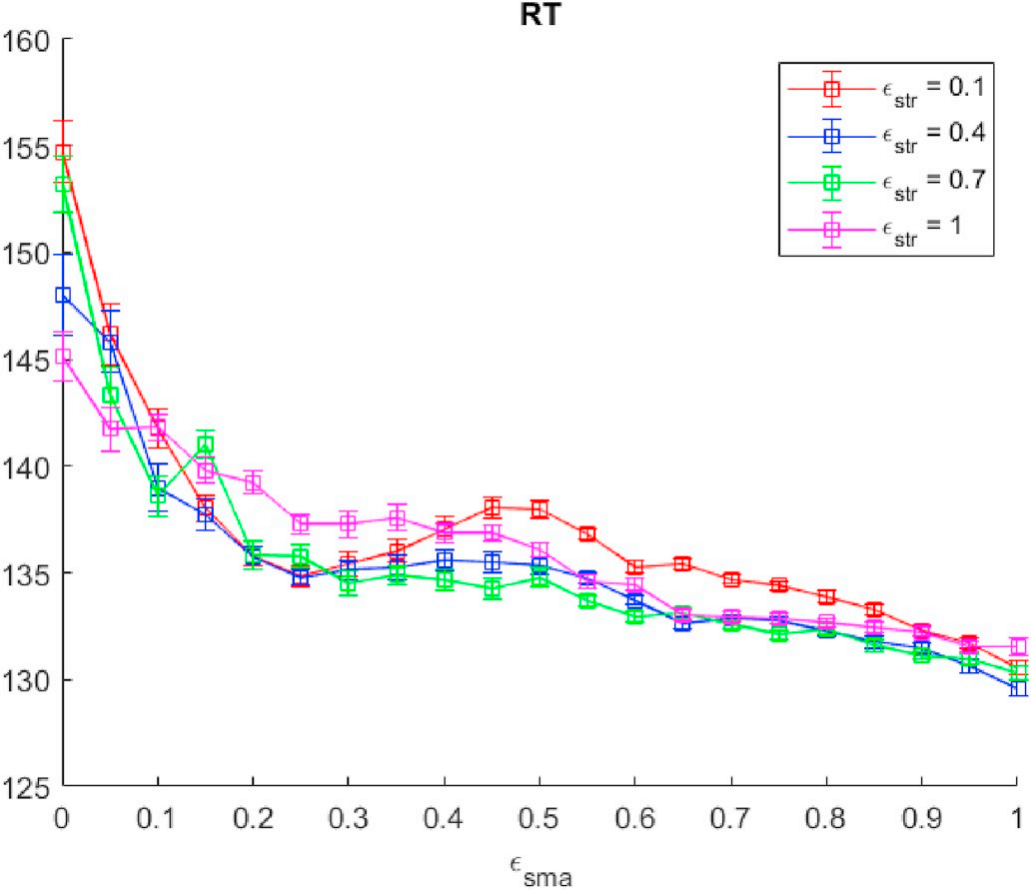
Response Time (in cycles) as a function of $\varepsilon_{sma}$ for four different values of $\varepsilon_{str}$. RT decreases as $\varepsilon_{sma}$ increases, but is largely independent of $\varepsilon_{str}$. Error bars show one standard error from the mean.

**Fig. 15:**
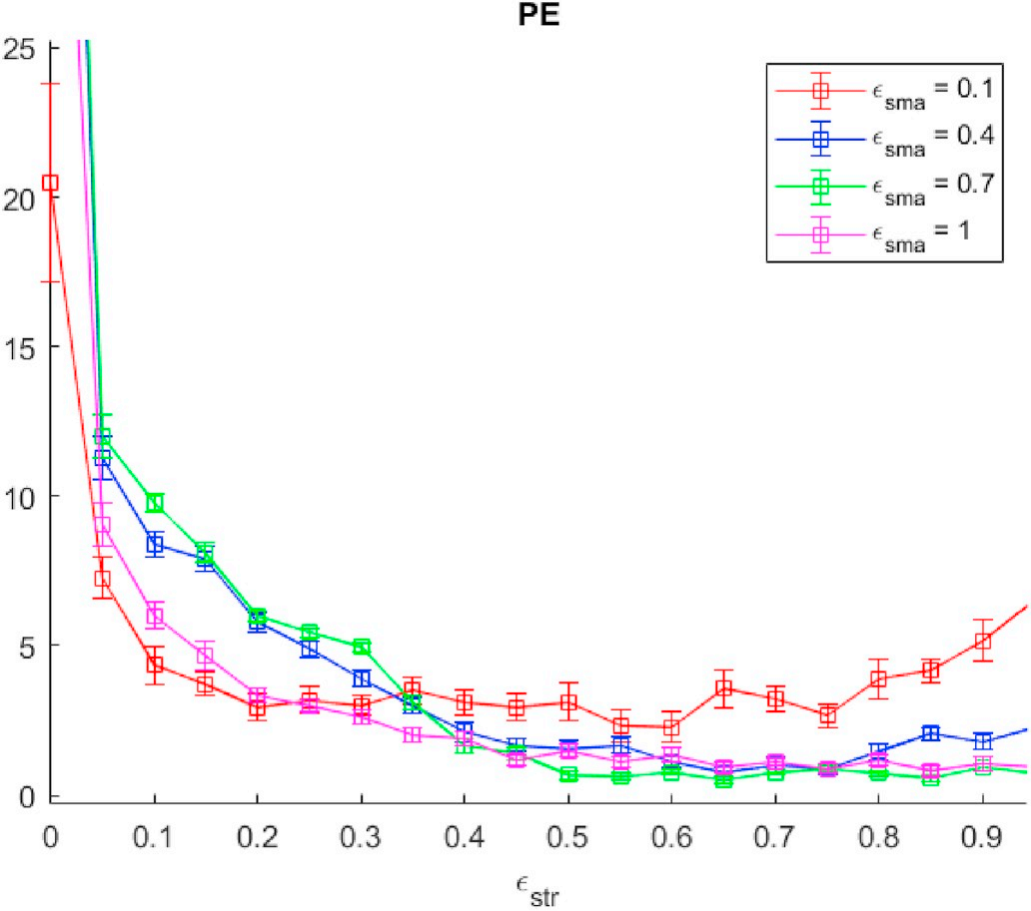
Perseverative Errors as a function of $\varepsilon_{str}$, for four different values of $\varepsilon_{sma}$. Note that perseverative errors are largely constant except at low values of $\varepsilon_{sma}$ or when both $\varepsilon_{sma}$ approaches 1 and $\varepsilon_{str}$ approaches 0. Error bars show one standard error from the mean.

**Fig. 16:**
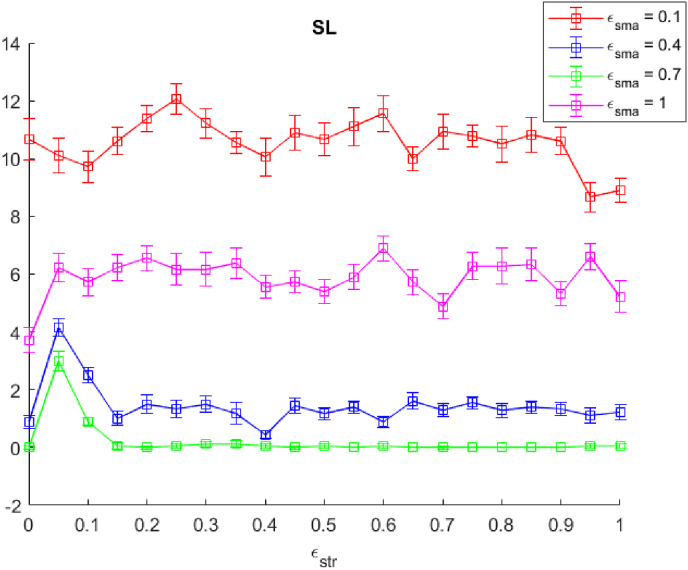
Set Loss errors as a function of $\varepsilon_{str}$, for four different values of $\varepsilon_{sma}$. Note that set loss errors are largely independent of $\varepsilon_{str}$, but are high at extreme values of $\varepsilon_{sma}$. When $\varepsilon_{sma}$ is 0.7 set loss errors are negligible except at very low values of $\varepsilon_{str}$. Error bars show one standard error from the mean.

**Fig. 17:**
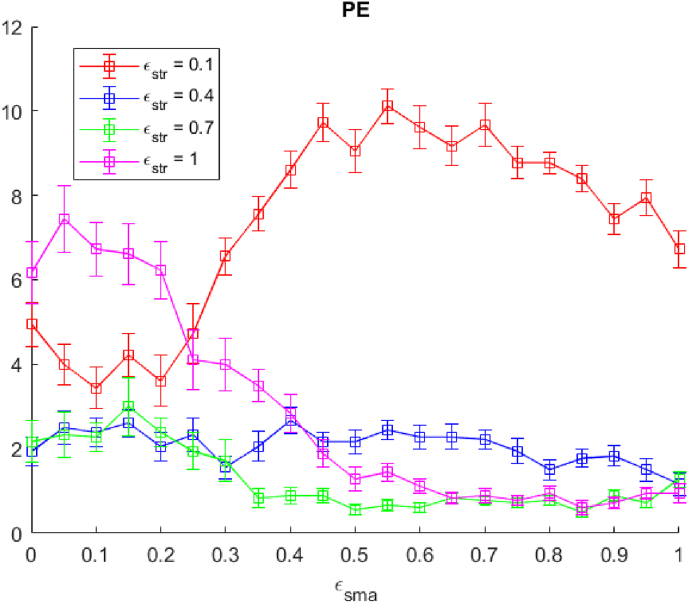
Perseverative Errors as a function of $\varepsilon_{sma}$, for four different values of $\varepsilon_{str}$. Consistent with Fig. 15, perseverative errors are infrequent except when $\varepsilon_{sma}$ is low or when $\varepsilon_{str}$ approaches 0. Error bars show one standard error from the mean.

**Fig. 18:**
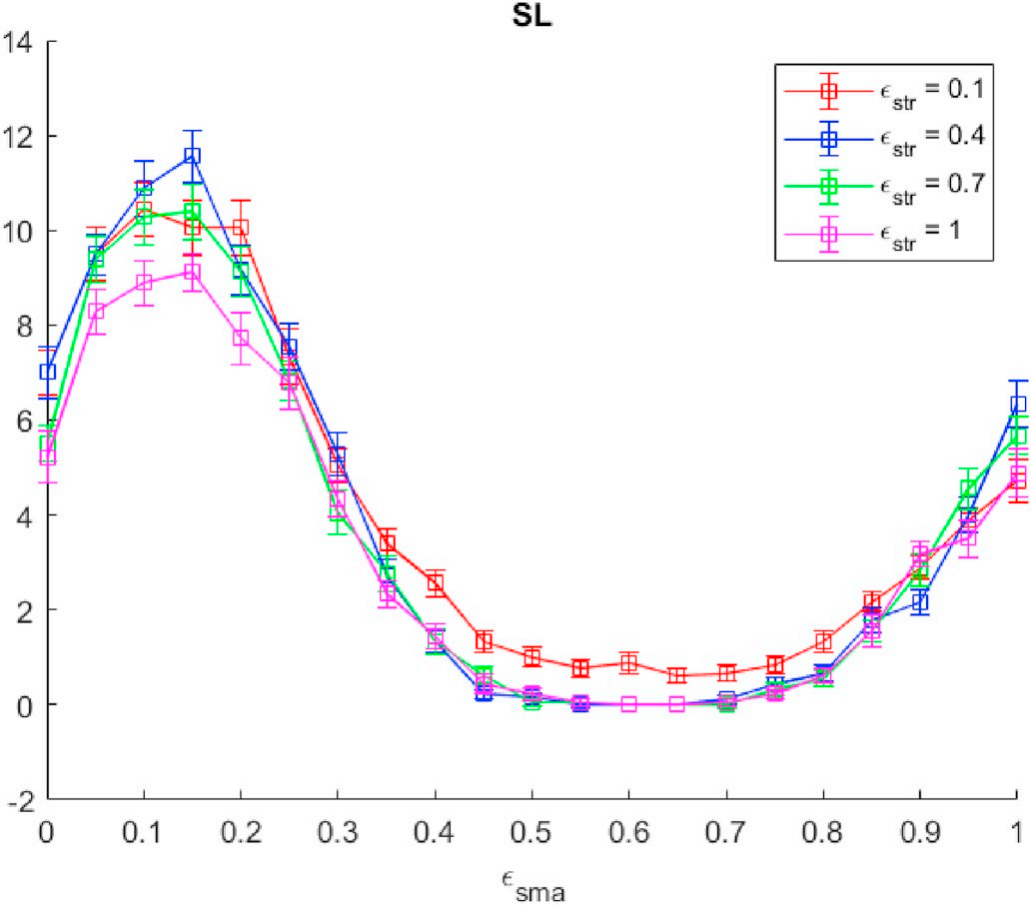
Set Loss errors as a function of $\varepsilon_{sma}$, for four different values of $\varepsilon_{str}$. Consistent with Fig. 16, set loss errors are largely independent of $\varepsilon_{str}$, but are high at extreme values of $\varepsilon_{sma}$. When $\varepsilon_{sma}$ is between 0.45 and 0.75 set loss errors are negligible except at very low values of $\varepsilon_{str}$. Error bars show one standard error from the mean.

**Fig. 19:**
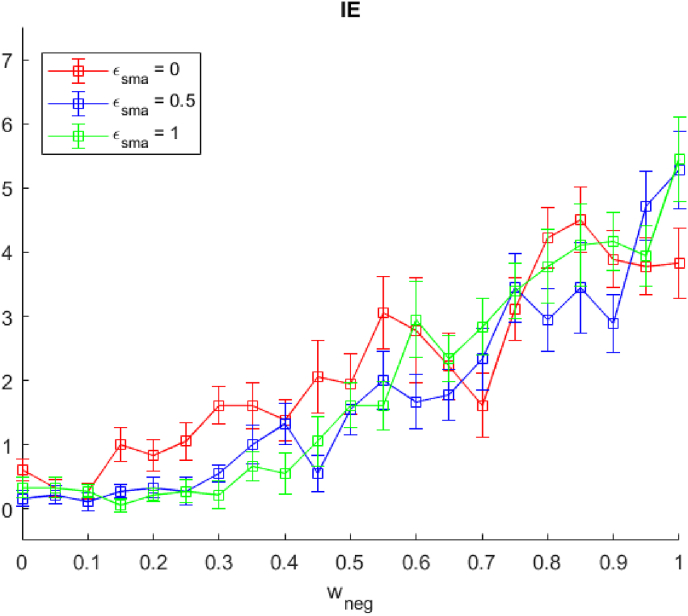
Integration Errors as a function of $w_{neg}$, for three values of $\varepsilon_{sma}$. Recall that $w_{neg}$ determines reward sensitivity in the striatum, with increasing values of $w_{neg}$ corresponding to decreasing reward sensitivity. Decreasing reward sensitivity results in an increased tendency to Integration Errors at all values of $\varepsilon_{sma}$. Error bars show one standard error from the mean.

**Fig. 20:**
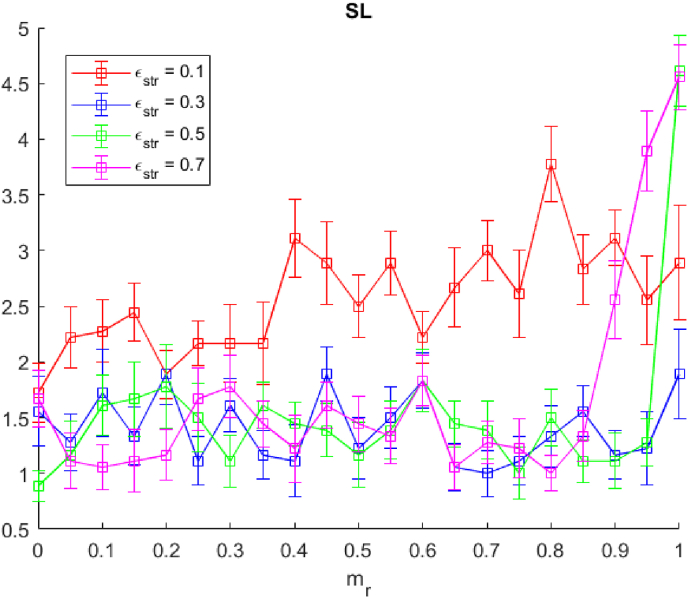
Set Loss Errors as a function of $m_{r}$, for four different values of $\varepsilon_{str}$. Error bars show one standard error from the mean.

**Fig. 21:**
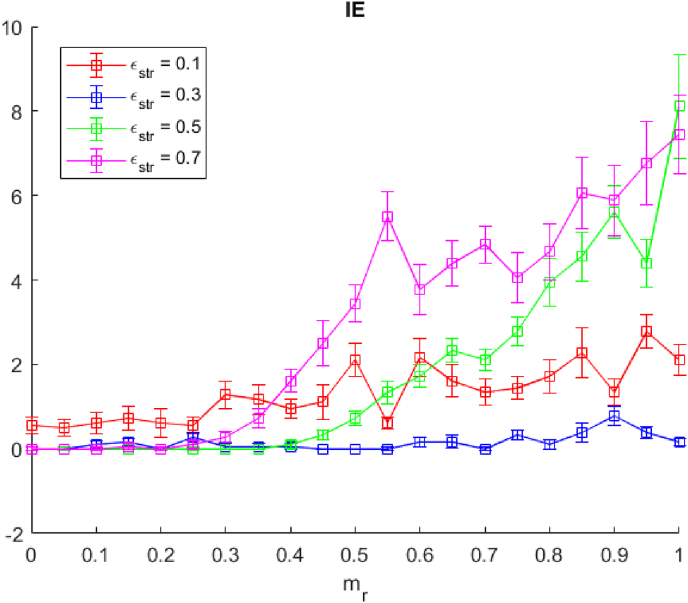
Integration Errors as a function of $m_{r}$, for four different values of $\varepsilon_{str}$. Error bars show one standard error from the mean.

## Appendix Sensitivity of the Model to Parameter Values

### Rationale

The model has a large number of parameters, as shown in Table 1. It is therefore important to demonstrate the extent to which the model's behaviour is dependent upon specific values of those parameters. At the same time, many parameters relate to the activation functions of each unit within the basal ganglia, which are independently parameterised. Moreover, many of the parameters are shared with the original model of Gurney et al. (2001). In order to analyse the qualitative behaviour of the model we therefore fix the values of the parameters shared with the original model to the values used in that original work, and focus on four key parameters: $\varepsilon_{str}$, $\varepsilon_{sma}$, $w_{neg}$, $m_{r}$. This appendix considers how variation in these critical parameters affects model performance.

### Method

The values of each of the critical parameters were systematically varied between 0 and 1 in increments of 0.05, and across either three or four values of each of the other critical parameters. For each point in parameter space 20 simulations (corresponding to 20 virtual participants) were run. Each simulation consisted of presentation of 64 unambiguous cards.

Roberts and Pashler (2000) have raised concerns about the ability of models to fit any dataset given an arbitrary number and range of parameters. While these concerns might not necessarily apply to qualitative modelling, it is nevertheless important to show that trends in model behaviour are the product of the model architecture rather than a specific set of parameter values. Therefore, in the simulations below a small amount of uniform noise (corresponding to a maximum variation of ±10% in each parameter's values) was also added to the values of all three parameters as well as to $w_{rule}$.^4^

### Results and Discussion

We explored the effects of joint variation of $\varepsilon_{str}$ and $\varepsilon_{sma}$ on response time and sorting errors. With respect to the former, as shown in Fig. 14, an increase in response time (RT) can be seen as either $\varepsilon_{str}$ or $\varepsilon_{sma}$ decreases. Unlike the steady decrease of RT with changes in $\varepsilon_{sma}$, a dramatic drop in RT occurs only when $\varepsilon_{str}$ falls below an approximate value of 0.2. Since $\beta_{str}$ can be associated with the amount of striatal dopamine, and the regulation of this parameter is driven by $\varepsilon_{str}$, these results are consistent with the appearance of PD motor symptoms after the destruction of a considerable proportion of neurons in the substantia nigra pars compacta (SNpc) (Cheng et al., 2010). Note that this does not require the introduction of any notion of plasticity to explain neurobiological compensatory mechanisms.

Turning to errors, reducing $\varepsilon_{str}$ increases the number of perseverative errors (see Fig. 15) but has no effect on set loss errors (see Fig. 16). Conversely, reducing $\varepsilon_{sma}$ has a noticeable effect on perseveration only when $\varepsilon_{str}$ is small (Fig. 17) and its relationship with set maintenance (as indicated by SL errors) is close to an inverted-U shape, except for smaller values of $\varepsilon_{sma}$ (Fig. 18). This suggests that there is an optimal level of conflict control, which is consistent with the idea that excessive control can interfere with processes where the environment (stimuli, here) provides the necessary level of information (Bocanegra and Hommel, 2014).

Importantly, as shown by Fig. 19, $\varepsilon_{sma}$ has a much smaller effect on information integration errors (IE) than $w_{neg}$. This parameter lessens negative reward sensitivity, and therefore impairs the search for the correct schema. For instance, a participant might receive negative feedback after sorting by colour, and then move to sorting by number. If negative feedback reception is impaired, it is more likely that the participant will return to sorting by colour again, committing an IE. This also establishes a direct relationship between reward sensitivity and schema memory, consistent with the observations in Davidow et al. (2016). For the same reasons, $w_{neg}$ has a very similar effect — of increasing the frequency of all other types of error, although to different extents.

The effect of increasing parameter $m_{r}$ is to increase persistence of feedback from the previous trial, both in terms of feature matching and in terms of previous reward (the two could potentially be dissociated). A value of $m_{r}$ above around 0.75 sharply increases Set Loss errors (see Fig. 20), while Integration Errors increase more gradually starting from $m_{r}$ of 0 (see Fig. 21). The dissociation is regulated by $\varepsilon_{str}$ and the interval where SL and IE do not correlate may well be regulated by $w_{neg}$. In summary, this parameter increases the exploration of new rules irrespective of feedback, but it regulates whether the exploration is directed more towards new states (higher SL) or old states (higher IE), therefore partially dissociating SL errors and IE, unlike other parameters in the model. It has been argued that the number of integration errors, but not the number of other types of errors, increases with age (Rhodes, 2004), suggesting that aging compromises rule inference (associated with IE) rather than set maintenance (associated with SL). This claim is consistent with the fact that weakened rule-inference may be linked to reduced working memory capacity in older individuals (Hartman et al., 2001). However, the $m_{r}$ parameter also suggests an alternative (or possibly complementary) explanation, namely that persistence of reward and feature-matching schemas can also account for integration errors. While this may seem paradoxical at first, it shows that increasing the saliency of competing representations increases exploration and therefore SL and IE.

Note that all three error types are mutually exclusive (i.e., an error cannot be both an IE and a PE, or an IE and an SL, or a PE and an SL), and so an increase in the frequency of one type of error tends to decrease the likelihood of occurrence of other types or error. This warrants caution in interpreting error values towards the boundaries. However, barring situations where one error type dominates (i.e., the boundaries), the relationship between the parameters and resultant error types is clearly complex and non-linear.

## Footnotes

1 Such higher-level schemas may be learned, as a consequence of automatising behavioural sequences, or constructed on the fly and temporarily maintained by the supervisory system, in response to the perceived need for deliberate control (see Shallice and Burgess, 1993), as might be required, for example, when confronted by roadworks when driving and hence when a change of route is required.

2 There are at least two further differences between the standard task and the so-called Madrid version of Barceló (2003). Firstly, in the Madrid version participants are told not whether their responses are “correct” or “incorrect”, but whether they should “repeat” or “shift” their sorting criterion. Thus, rule shifting is prompted not by participants making an error but by participants being explicitly told to shift rules. Secondly, the Madrid version features frequent rule shifts — on average after 3 to 4 trials, while in the standard version rules are maintained for 10 trials. The model presented here assumes the standard presentation of feedback and the standard frequency of rule shifts.

3 Pairwise comparisons indicate that these effects are all statistically significant, but such comparisons are not particularly meaningful given that their results can be manipulated by manipulating the simulated sample sizes.
